# DNA demethylating agents suppress preclinical models of synovial sarcoma

**DOI:** 10.1172/JCI190855

**Published:** 2025-04-29

**Authors:** Nobuhiko Hasegawa, Nezha S. Benabdallah, Kyllie Smith-Fry, Li Li, Sarah McCollum, Jinxiu Li, Caelen A. Jones, Lena Wagner, Vineet Dalal, Viola Golde, Anastasija Pejkovska, Lara Carroll, Malay Haldar, Seth M. Pollack, Scott W. Lowe, Torsten O. Nielsen, Ana Banito, Kevin B. Jones

**Affiliations:** 1 Department of Orthopaedics,; 2 Department of Oncological Sciences,; 3 Huntsman Cancer Institute, University of Utah, Salt Lake City, Utah, USA.; 4 Department of Orthopaedics, Juntendo University, Faculty of Medicine, Tokyo, Japan.; 5 Hopp Children’s Cancer Center, Heidelberg (KiTZ) Heidelberg, Germany.; 6 German Cancer Research Center (DKFZ), Heidelberg, Germany.; 7 National Center for Tumor Diseases (NCT), NCT Heidelberg, Heidelberg, Germany.; 8 Edinburgh Cancer Research, CRUK Scotland Centre, Institute of Genetics and Cancer, University of Edinburgh, Edinburgh, United Kingdom.; 9 Department of Pathology and Laboratory Medicine, Perelman School of Medicine at the University of Pennsylvania, Philadelphia, Pennsylvania, USA.; 10 Department of Medicine, Feinberg School of Medicine at Northwestern University, Chicago, Illinois, USA.; 11 Cancer Biology and Genetics Program, Memorial Sloan-Kettering Cancer Institute, New York, New York, USA.; 12 Howard Hughes Medical Institute, Chevy Chase, Maryland, USA.; 13 Department of Pathology and Laboratory Medicine, Vancouver Coastal Health Research Institute and Faculty of Medicine, University of British Columbia, Vancouver, British Columbia, Canada.

**Keywords:** Genetics, Oncology, Cancer, Epigenetics, Expression profiling

## Abstract

Synovial sarcoma is an aggressive soft-tissue cancer driven by the chimeric SS18::SSX fusion oncoprotein, which disrupts chromatin remodeling by combining two antagonistic transcriptional regulators. SS18 participates in BAF complexes that open chromatin, while the SSX genes are cancer-testis antigens that interface with chromatin decorated with monoubiquitinated histone H2A placed by polycomb repressive complex activity. Because KDM2B brings polycomb repressive complex to unmethylated CpG islands, it is plausible that methylation directly determines the distribution of SS18::SSX to target loci. Given that synovial sarcoma is also characterized by a peculiarly low DNA hypomethylation profile, we hypothesized that further disturbance of DNA methylation would have a negative impact on synovial sarcoma growth. DNMT1 disruption by CRISPR/Cas9 targeting or pharmacological inhibition with cytidine analogs 5-aza-2′-deoxycytidine (decitabine) and 5-azacytidine led to decreased genome-wide methylation, redistribution of SS18::SSX, and altered gene expression profiles, most prominently including upregulation of tumor suppressor genes, immune-related genes, and mesenchymal differentiation-related genes. These drugs suppressed growth of synovial sarcoma cell lines and drove cytoreduction in mouse genetic models. DNMT1 inhibitors, already approved for treating myelodysplastic syndromes, warrant further clinical investigation for synovial sarcoma as repurposed, targeted treatments exploiting a vulnerability in the intrinsic biology of this cancer.

## Abstract 2

Synovial sarcoma is a deadly malignancy charcaterized by hypomethylation of CpG islands in gene regulatory elements genome-wide. Hypomethylating drugs that push this aberrant biology further are successful in treating this disease.

## Graphical abstract

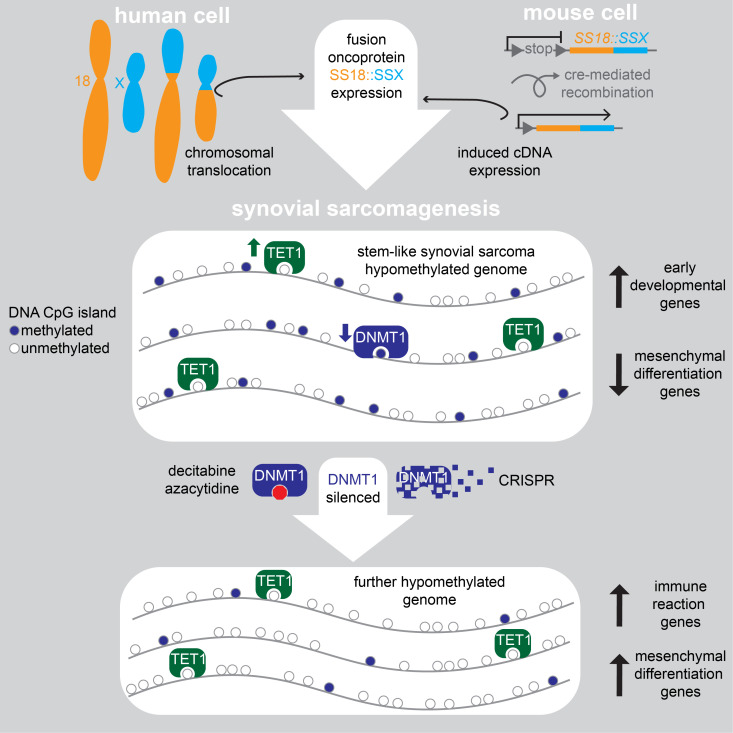

## Introduction

Synovial sarcoma is a soft-tissue sarcoma most frequently diagnosed in adolescents and young adults; one-third of all cases occur in patients under the age of 20 (1). Current treatment strategies consist primarily of localized surgical resection often supported by (neo)adjuvant radiation therapy (2). Although systemic treatments, such as doxorubicin, ifosfamide, trabectedin, or pazopanib, are also frequently administered, they have limited impact on patient survival rates, which are poor after the development of progressive disease and metastasis (3). Despite these relatively poor treatment outcomes, the field of synovial sarcoma therapies has not drastically affected disease survivability in the past 30 years. Although the cellular therapy afamitresgene autoleucel was recently approved for synovial sarcoma, only a fraction of patients are eligible for this therapy, complete responses were very rare, and only a minority of patients experienced durable overall responses, all at the cost of high toxicity (4). This limited progress highlights the continuing need for innovative therapeutics in synovial sarcoma treatment, particularly any that can target the unique biology underlying this cancer of young patients.

Synovial sarcoma belongs to the category of fusion gene-driven sarcomas, being defined by the chromosomal translocation t(X;18) (p11.2;q11.2), which leads to the formation of a fusion oncogene from which is expressed the fusion oncoprotein SS18::SSX (5, 6). This chimeric oncoprotein alone is sufficient to drive sarcomagenesis in the mouse, and is associated with a very low tumor mutational burden in humans and mice (7–12). SS18, the amino-terminal partner of this fusion protein, is a member of BRG1/BRM and associated factors (BAF) complexes, which are important chromatin remodelers involved in cell stemness and differentiation (13). The contributor of the carboxy terminus varies slightly, encoded by translocation to *SSX1*, *SSX2*, or, rarely, *SSX4* (14, 15). The SSX proteins are considered cancer-testis antigens, whose wild-type expression is often associated with spermatogenesis or malignancies (16–18). Among other potential mechanisms of action, the SSX carboxy termini interact with nucleosomes marked by histone posttranslational modifications placed by polycomb repressive complexes, a family of important chromatin regulators. Upon expression of SS18::SSX, the fusion protein replaces wild-type SS18, becoming incorporated into the noncanonical GBAF (GLTSCR1-containing BAF) and canonical BAF complexes (19, 20). These events have major consequences, redirecting the BAF complex within the genome to alter transcription, ultimately resulting in tumorigenesis (19). Although the driving mutation of synovial sarcoma is well characterized, the resulting fusion oncoprotein is not yet readily targetable with therapy. This renders any opportunity to target fusion-adjacent biology as an attractive potential therapeutic option.

We and others have recently shown that SS18::SSX is recruited to chromatin via its interaction with nucleosomes bearing monoubiquitinated lysine 119 of histone H2A (H2AK119ub), placed by PRC1.1 (21–23). Lysine demethylase 2 (KDM2B; also known as FBXL10 or JHDM1B) is the component of PRC1.1 responsible for loading the complex at precise genomic locations by recognizing and binding unmethylated CpG islands through its ZF-CxxC domain (24–26). KDM2B then drives the recruitment of other PRC1.1 components (BCOR, PCGF1, and RING1B/RNF2) to chromatin, placing H2AK119ub (26–28). Synovial sarcoma has also been characterized as generally hypomethylated across its genome (11), and particularly at promoter sites (29).

Methylation inhibitors, including the cytidine analogs 5-aza-2′-deoxycytidine (decitabine) and 5-azacytidine (5-AZA), have long been utilized in the treatment of hematological malignancies, such as myelodysplastic syndrome (30). The mechanisms of antineoplastic activity of demethylating agents are wide-ranging, including effects on apoptosis, differentiation, angiogenesis, senescence, and other processes (31).

In many of their most efficacious uses, demethylating agents have induced the re-expression of genes, such as tumor-suppressor genes or neoantigens that were silenced by promoter methylation in the cancer cells (32). We speculated that the use of these drugs in synovial sarcoma may affect tumor development by further broadening and diluting the distribution of KDM2B and thus redistributing the SS18::SSX fusion oncoprotein to newly hypomethylated CpG islands, or by hypomethylating and activating the few genes that retain promoter hypermethylation as a means of silencing tumor suppressors or neoantigens recognized by the immune system.

## Results

### Synovial sarcoma exhibits a dependency on DNMT1.

Synovial sarcomas display a striking promoter DNA hypomethylation in which synovial sarcoma displays the lowest mean methylation level of any sarcoma. Importantly, this hypomethylation is specific to promoter regions and is not observed genome wide, in gene bodies or intergenic regions (29). Interestingly, SS18::SSX has a known relationship with methylation profiles as its chromatin binding depends on the presence of KDM2B, the member of the polycomb repressive complexes that recognizes and binds unmethylated CpG islands (21). We therefore hypothesized that synovial sarcoma might be particularly sensitive to the removal of enzymes that regulate DNA methylation.

**Figure 1:**
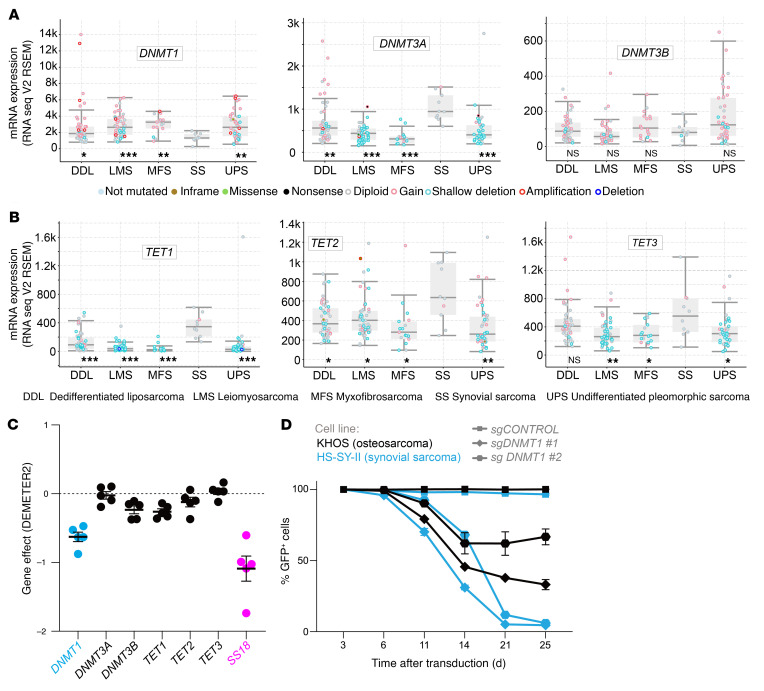
The synovial sarcoma genome is hypomethylated by increased TET and decreased DNMT1 expression. (**A**) *DNMT1*, *DNMT3A*, and *DNMT3B* mRNA expression and mutational profile from cBioPortal analysis. Expression levels for 20,532 genes in 264 soft-tissue cases (RNA Seq V2 RSEM). GISTIC 2.0; –2 = deep deletion; –1 = shallow deletion; 0 = diploid; 1 = gain; 2 = amplification. *P* values were determined by Mann-Whitney *U* test. *P < 0.05, **P < 0.005, ***P < 0.0005; ns, not significant. (**B**) *TET1, TET2*, and *TET3* mRNA expression and mutational status. (**C**) Gene-specific dependence scores for each synovial sarcoma cell line in the Target Drive initiative accessed by DepMap. (**D**) Cell competition assay performed in the osteosarcoma cell line KHOS-240S-Cas9 (fusion negative control) or in the synovial sarcoma line HS-SY-II-Cas9 transduced with a safe sgRNA as control or with guides targeting DNMT1.

We first investigated whether the genes that regulate DNA methylation (*DNMT1, DNMT3A, DNMT3B, TET1, TET2, TET3)* are misexpressed, mutated, or amplified in synovial sarcoma. We performed in silico analysis using cBioPortal and DepMap public databases and compared mRNA expression levels and putative copy-number alterations among The Cancer Genome Atlas (TCGA) dataset of human soft-tissue sarcomas: dedifferentiated liposarcoma (50 cases), leiomyosarcoma (53 cases), myxofibrosarcoma (17 cases), synovial sarcoma (10 cases), and undifferentiated pleomorphic sarcoma (44 cases). In synovial sarcoma, the mRNA expression levels of *DNMT1* and *DNMT3B* were similar to or lower than those in other sarcomas, whereas *DNMT3A* exhibited higher expression levels (Figure 1A). Expression levels of *TET* family genes that drive DNA demethylation were also higher in synovial sarcoma (Figure 1B) compared with other sarcoma subtypes. This corroborates recent findings showing elevated expression of *TET1* in human and murine synovial sarcoma when compared with normal cell counterparts (29). We did not observe prevalent copy number alterations or mutations in *DNMT* or *TET* genes in synovial sarcoma.

Given that higher levels of *TET1* expression are not associated with a dependency in synovial sarcoma (29), we sought to probe whether other enzymes are vulnerabilities. Using DepMap’s RNA interference (RNAi) data across synovial sarcoma cell lines and the genes that orchestrate precise DNA methylation patterns, we observed that *DNMT1* is the only enzyme that displayed partial dependency, with a gene effect (Chronos) value approaching −1, which is comparable to the median of all pan-essential genes (Figure 1C). We further confirmed that ChIP-Seq data from our mouse genetic model identified the characteristic pattern of histone marks and fusion peaks around the *Tet3* locus that indicate it is directly targeted by the fusion oncoprotein (33), with similar — but less pronounced — marking of *Tet1* and *Dnmt3a* (Supplemental Figure 1; supplemental material available online with this article; https://doi.org/10.1172/JCI190855DS1). *Dnmt1*, *Dnmt3b*, and *Tet2* appear to be expressed with more of a housekeeping gene pattern of histone marks, less targeted by the fusion oncoprotein itself (Supplemental Figure 1).

CRISPR/Cas9 knockout of *DNMT1* using sgRNA (34) had a more negative impact on the growth of a synovial sarcoma cell line (HS-SY-II) compared with an osteosarcoma cell line (KHOS) (Figure 1D). Although *sgDNMT1* strongly depleted synovial sarcoma cells over time, the slow pace of depletion is consistent with the requirement for passive loss of DNA methylation with each cell division in cancer cell lines that have relatively long doubling times.

### Decitabine or DNMT1 genetic disruption induces a mesenchymal-like phenotype.

To evaluate the nature of the phenotypes caused by DNMT1 removal in synovial sarcoma, we targeted DNMT1 in synovial sarcoma human cell lines using sgRNA or decitabine, a cytidine analog that cannot be methylated and, after incorporation into DNA, progressively dilutes methylation markers in the chromatin of proliferating cells. Cells were evaluated at day 12 after *sgDNMT1* expression or decitabine treatment, allowing enough time for passive loss of DNA methylation.

**Figure 2:**
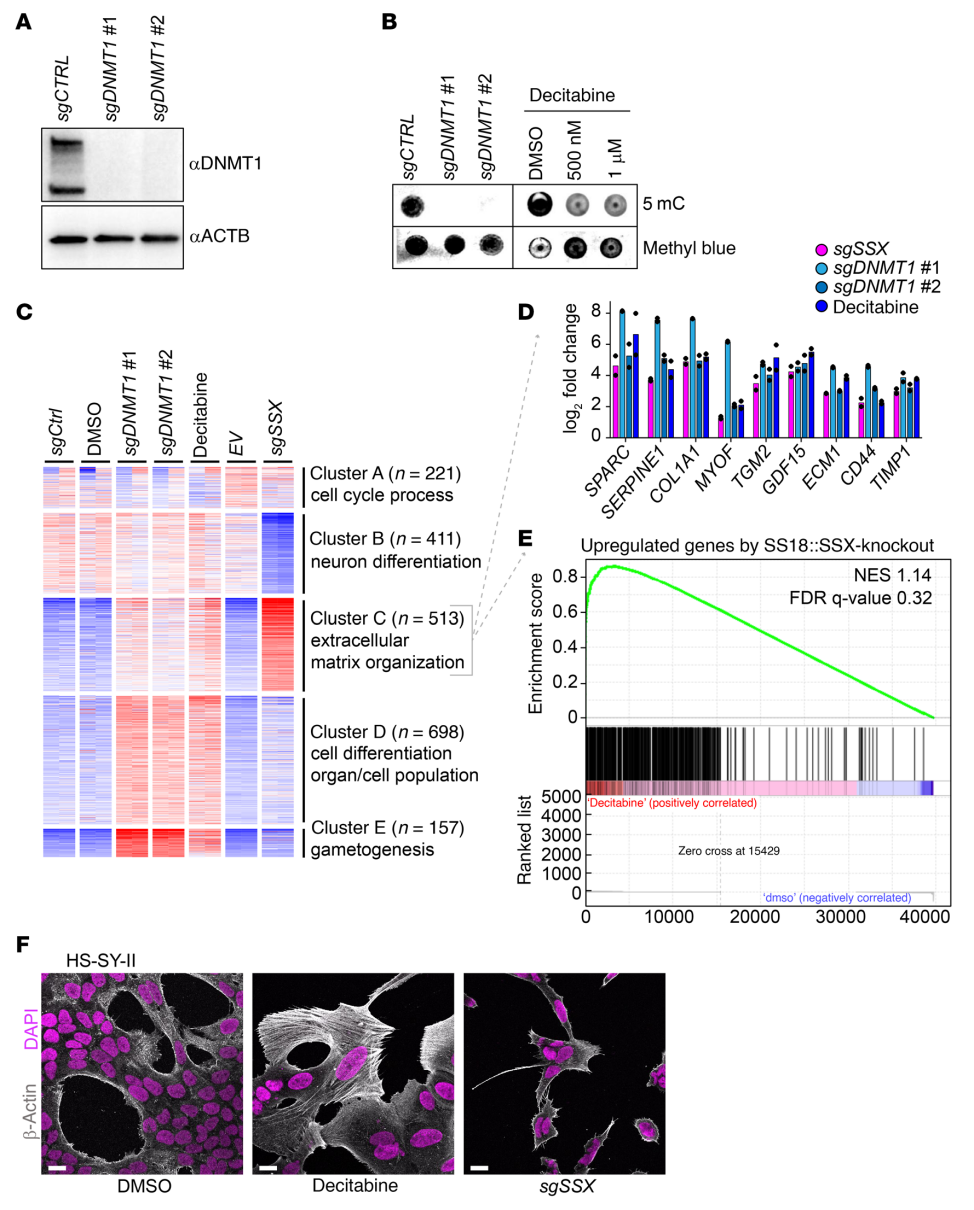
Decitabine treatment mimics *DNMT1* knockout and leads to mesenchymal-like phenotype. (**A**) Western blot of whole cell extract from HS-SY-II-Cas9 expressing either a safe sgRNA (CTRL) or sgRNAs targeting DNMT1 for 12 days revealed with either anti-DNMT1 or anti–β-actin antibodies. (**B**) Global methylation levels measured by 5mC methylation dot blot using 1 μg genomic DNA and methyl blue as loading control. (**C**) Heatmap of transcriptional analysis showing k-means clustering of the 2,000 most variable genes with a cutoff *z* score of 4 in HS-SY-II-Cas9–expressing safe sgRNA (CTRL) or targeting DNMT1 or HS-SY-II treated with either DMSO or 500 nM decitabine for 12 days. *n* = 2. (**D**) Log_2_ fold change of FPKM values from genes present in cluster C, extracellular matrix organization. Data represent the mean of 2 biological replicates. (**E**) Gene set enrichment analysis (GSEA) comparing the expression of the 513 genes present in cluster C with the top 500 genes upregulated upon SS18-SSX knockout. (**F**) Immunofluorescence of human synovial sarcoma HS-SY-II cells stained with DAPI (magenta) and β-actin (gray). Scale bar: 20 μm.

We first confirmed knockout efficiency by assessing DNMT1 levels, showing an evident reduction upon transfection of sgRNA against *DNMT1* (Figure 2A). DNMT1 disruption or inhibition using 500 nM or 1 μM decitabine led to a global decrease in 5-methylcytosine levels (Figure 2B).

Changes in methylation status can be monitored by evaluating the expression of certain methylation-sensitive genes, where promoter demethylation is followed by expected gene upregulation: *BCAS1, DAPK1, DNAH3, DNAH12,* and *ERC1* (35). We found that 500 nM decitabine treatment was sufficient to recapitulate the *DNMT1* disruption phenotype in this gene set (Supplemental Figure 2A). Additionally, RNA-Seq results revealed similar expression profiles in cells treated with 500 nM decitabine compared with *DNMT1* disruption, indicating that the decitabine treatment had limited off-target effects (Supplemental Figure 2B). Collectively, these results suggest that 500 nM decitabine substantially decreased global methylation levels and mimicked *DNMT1* disruption.

Gene expression was evaluated after treatment with decitabine, *sgDNMT1*, and *sgSSX*, as well as corresponding controls. The 5 major groups were divided into clusters A–E (Figure 2C). In cluster C, we selected genes with a log_2_ fold-change of fragments per kilobase of exon per million mapped fragments (FPKM) values greater than 0.5 compared with their controls and confirmed the overlap of *sgSSX*, *sgDNMT1*, and decitabine treatment. The other 2 groups were subsumed into the *sgSSX* group of upregulated expression genes (Supplemental Figure 2C). Comparison of *SS18::SSX* disruption to decitabine administration revealed that genes whose expression was downregulated by either had minimal overlap (Supplemental Figure 2, D and E), whereas the genes upregulated in *SS18::SSX* disruption were also upregulated after decitabine treatment (Figure 2D). Several genes were commonly upregulated in *sgDNMT1* and decitabine treatments in cluster C, with increased expression in *sgSSX* (Figure 2E). This cluster was enriched in extracellular matrix organization genes and other mesenchymal genes known to be expressed in fibroblasts. Accordingly, decitabine treatment triggered proliferative arrest and the acquisition of a fibroblast-like morphology in a manner that was substantially similar to the depletion or genetic disruption of *SS18::SSX1* (21). SS18::SSX function in mesenchymal lineage determination is likely partly mediated by DNA methylation of differentiation gene promoters. In HS-SY-II and SYO-1 cells, both decitabine administration and *sgSSX* showed similar impacts on cell morphology (Figure 2F and Supplemental Figure 2F). These observations indicate that reduced DNMT1-mediated genome methylation phenocopies some changes in synovial sarcoma morphology and cell growth associated with SS18::SSX depletion.

### Synovial sarcoma cells exhibit sensitivity to decitabine and 5-AZA.

**Figure 3:**
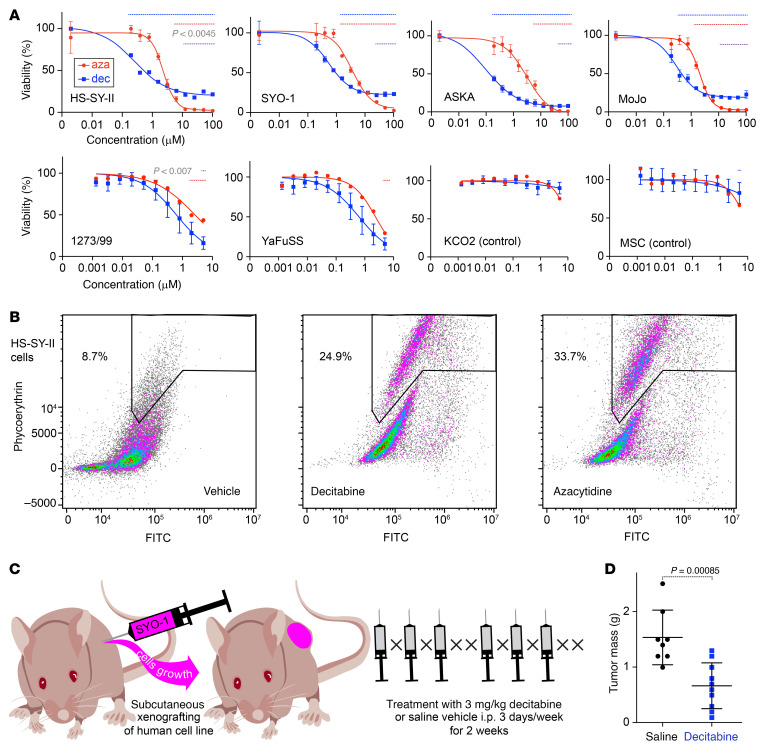
Synovial sarcoma is sensitive to decitabine and azacytidine. (**A**) Viability of 6 synovial sarcoma cell lines and 2 untransformed mesenchymal cell lines treated with decitabine or 5-AZA. The blue dotted line indicates the points where the *P* value of the cell survival rate compared with the reference point in decitabine is significant. The red dotted line indicates the comparison to the reference for 5-AZA. (1273/99, YaFuSS, KCO2, and MSC are compared based on pooling of the initial 2 points.) The purple dotted line indicates the significantly different regions in each point of decitabine and 5-AZA in HS-SY-II, SYO-1, ASKA, and MoJo. In MoJo, outlier values of more than 5 times the decitabine at 100 μM were excluded. *P* values were determined by 2-tailed paired *t* tests. (*P* values of less than 0.0045 were considered significant in the upper row, less than 0.007 for the lower row, each Bonferroni corrected from 0.05). (**B**) Necrosis areas similarly framed for control (vehicle), decitabine-treated, and 5-AZA–treated groups. (**C**) Diagram of a xenograft mouse model and details of drug administration. (**D**) Comparison of tumor mass after 2 weeks of treatment. *P* value was determined by 2-tailed homoscedastic *t* test. (*n* = 8 control, *n* = 10 decitabine treatments).

To determine whether DNMT1-mediated methylation landscape reprogramming can be used as a therapeutic strategy in synovial sarcoma, we performed a drug sensitivity test using decitabine or 5-AZA in a panel of different synovial sarcoma cell lines, with 2 untransformed mesenchymal cell lines (KCO2 or MSCs) as controls (Figure 3A). All 6 synovial sarcoma cell lines (HS-SY-II, SYO-1, ASKA, MoJo, 1273/99, and YaFuSS) exhibited lower 50% IC_50_ values for decitabine and 5-AZA when compared with untransformed control cells. Additionally, IC_50_ levels were lower for decitabine when compared with 5-AZA in synovial sarcoma lines, but at higher concentrations, 5-AZA exhibited a stronger cytotoxic effect than decitabine (Figure 3A). We compared HS-SY-II cells that were untreated or treated with IC_50_-guided concentrations of decitabine (500 nM) and 5-AZA (3 μM) for 6 days and measured apoptosis and necrosis using a caspase-3/7 green flow cytometry assay. Both treatments resulted in induction of apoptosis and necrosis, and 5-AZA exhibited a slightly higher necrosis percentage (Figure 3B). Finally, for an in vivo correlate of human cell line growth, we tested the SYO-1 cell line xenografted into the subcutaneous flanks of immunocompromised host mice (Figure 3C). Tumors harvested after treatment with decitabine were smaller than those treated with saline vehicle (Figure 3D and Supplemental Figure 3A). In histomorphology on H&E-stained sections of the xenograft tumors, the decitabine treatment group showed more spindled cell shapes and more collagenous matrix production, as further demonstrated on Masson’s trichrome stain (Supplemental Figure 3, B and C). These results indicate that synovial sarcoma is sensitive to hypomethylation agents, which, in addition to growth arrest and induction of mesenchymal-related genes, trigger at least some degree of growth inhibition by cell death.

### Transcriptomic analysis of human synovial sarcoma cell lines treated with demethylases.

**Figure 4:**
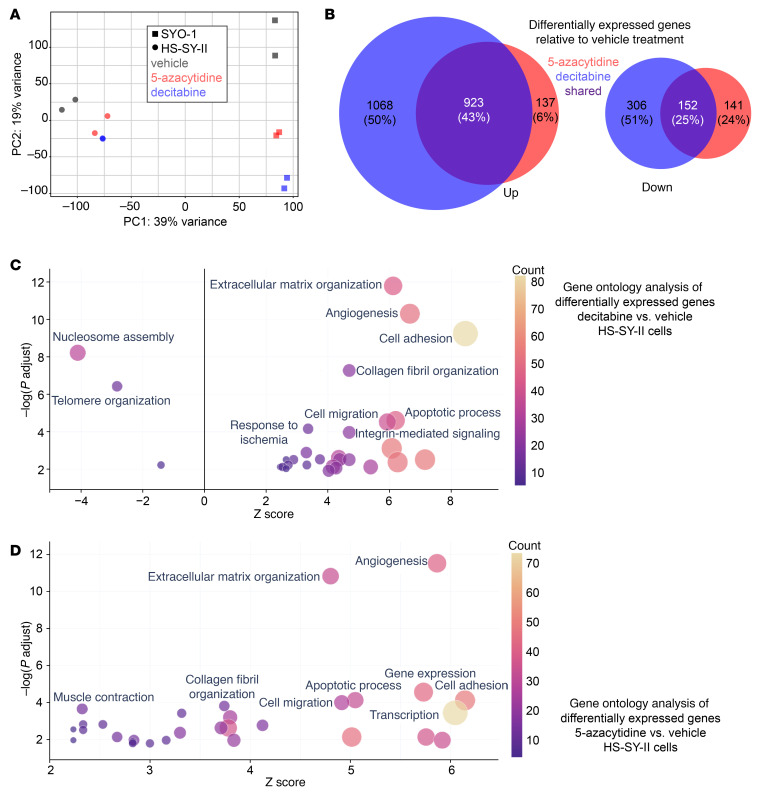
Transcriptomes change from decitabine or azacytidine applied to human synovial sarcoma cell lines. (**A**) TPM-based principal component analysis of whole transcriptomes. (**B**) Genes with expression variation log_2_ fold-change expression (≤1/>1) and adjusted *P* < 0.05; (left) group with increased expression, (right) group with decreased expression. (**C**) Bubble plots from GO analysis of upregulated and downregulated genes in HS-SY-II cells after decitabine treatment compared with vehicle. (**D**) GO analysis of upregulated genes and the biological processes in HS-SY-II cells after 5-azacytidine treatment.

To further explore the mechanisms by which decitabine and 5-AZA inhibit tumor growth, we evaluated their effects on the transcriptome in HS-SY-II and SYO-1 cell lines. A principal component analysis plot using transcripts per million (TPM) distinguished cell lines and treatments versus controls (Figure 4A). In HS-SY-II, decitabine treatment upregulated 1,991 genes and downregulated 458 genes; 5-AZA treatment upregulated 1,060 genes and downregulated 293 genes. A total of 923 upregulated genes were shared between the decitabine and 5-AZA treatment groups, each relative to the control, along with 152 shared downregulated genes (Figure 4B). In SYO-1, decitabine treatment upregulated 1,498 genes and downregulated 1,341 genes; 5-AZA treatment upregulated 593 genes and downregulated 467 genes. A total of 533 upregulated genes were shared between the decitabine and 5-AZA groups, along with 415 downregulated genes (Supplemental Figure 4A). From Gene Ontology (GO) analysis for each group, we constructed bubble plots by selecting the top 30 terms with the lowest *P* values. Notably, in both cell lines treated with decitabine or 5-AZA, GO terms related to nucleosome assembly were decreased, whereas those associated with extracellular matrix organization were increased (Figure 4, C and D, and Supplemental Figure 4B).

### Decitabine and 5-AZA suppress tumor growth in synovial sarcoma mouse models.

**Figure 5:**
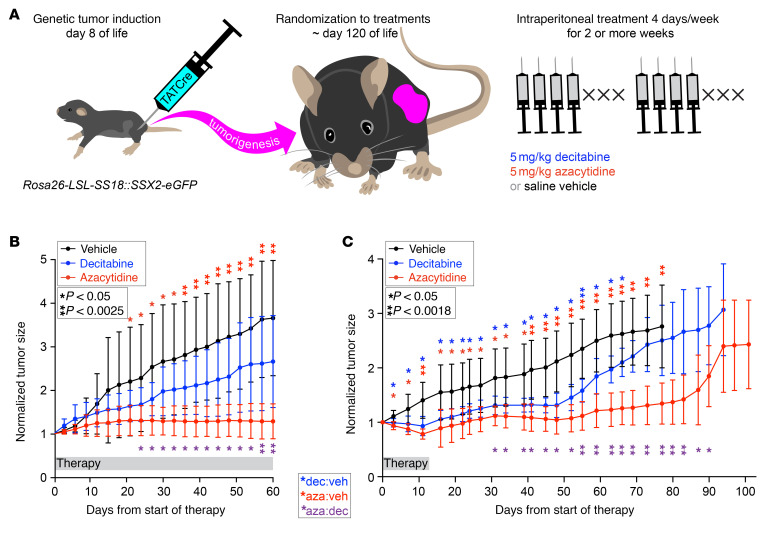
Decitabine and azacytidine treatments affect genetically induced synovial sarcomas in mice. (**A**) Diagram of a genetically induced mouse model and details of drug administration. (**B**) Tumor growth curves for each group. Normalized (fractional) tumor volumes are presented as mean ± SD. Two-tailed homoscedastic *t* test *P* values less than 0.05 are indicated with a single asterisk, those less than the Bonferroni-corrected *P* values are indicated with a double asterisk (red for 5-AZA compared with vehicle, purple for 5-AZA compared with decitabine; *n* = 8 for vehicle and decitabine treatments, and 10 for 5-AZA treatments). (**C**) Course of normalized mean tumor size from day 0 of treatment, administered for 14 days and then stopped. Two-tailed homoscedastic *t* test; *P* values less than 0.05 are indicated with a single asterisk, those less than the Bonferroni-corrected *P* value are indicated with a double asterisk (blue for decitabine compared with vehicle, red for 5-AZA compared with vehicle, purple for 5-AZA compared with decitabine; *n* = 9 for 5-AZA and decitabine treatment groups, and 5 for the vehicle control group).

We next evaluated the effects of hypomethylating agents in synovial sarcoma tumors genetically induced in mice with conditional expression of the *SS18::SSX2* fusion gene (Figure 5A) (9). Mice were treated with decitabine, 5-AZA, or saline vehicle for 2 months and tumor growth was monitored by caliper measurements. Both agents suppressed tumor growth (5-AZA significantly), even achieving cytoreduction of some tumors (Figure 5B). Treatment for 2 weeks with decitabine or 5-AZA also suppressed tumor growth for the subsequent 40 days after cessation of therapy; the decitabine group exhibited a rapid increase in tumor size thereafter. In contrast, tumor growth suppression persisted for approximately 80 days in the 5-AZA group after this brief treatment (Figure 5C).

### Effects of decitabine and 5-AZA on the transcriptome and methylome in mouse synovial sarcomas.

**Figure 6:**
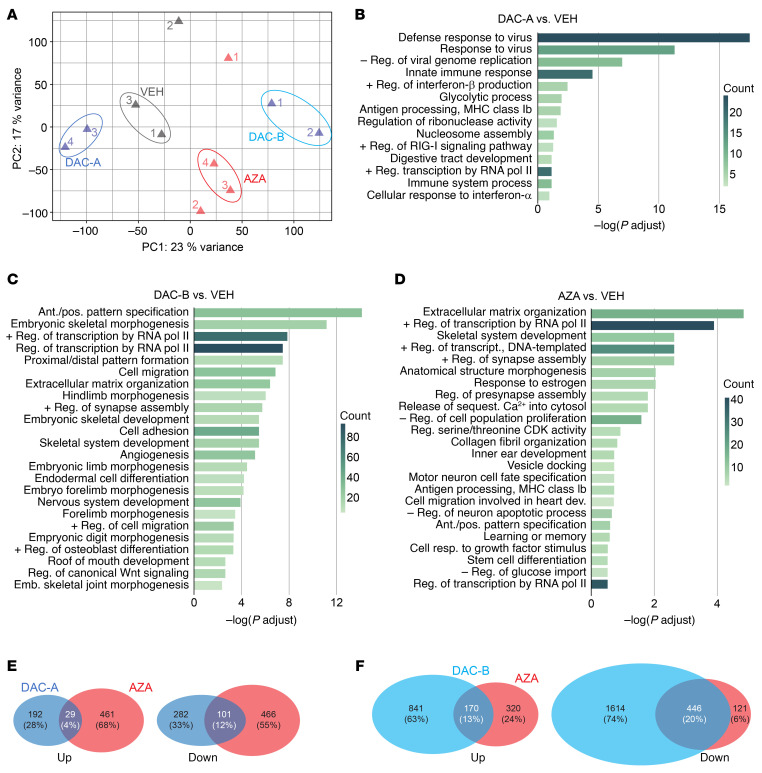
Transcriptional changes by pharmacological demethylation in genetically induced mouse synovial sarcomas. (**A**) TPM-based principal component analysis**.** Decitabine samples were classified by histology into 2 groups, DAC-A and DAC-B. (**B**) GO terms for biological processes for genes with upregulated differential expression log_2_ fold-change (≤1/>1) and adjusted *P* less than 0.05 in DAC-A compared with VEH group. (**C**) GO analysis of the biological processes in DAC-B over VEH upregulated genes. (**D**) GO analysis of the biological processes in genes upregulated in AZA group tumors compared with VEH. (**E**) Venn diagrams of shared upregulated and downregulated genes each relative to VEH of DAC-A and AZA groups. (**F**) Venn diagrams for shared genes between DAC-B and AZA, each compared with VEH.

RNA-Seq was also performed on tumors harvested after treatment in mice. A principal component analysis map based on TPM showed separation based on histopathology subtyping. Among the vehicle-treated samples, 1 and 3 were both biphasic/poorly differentiated tumors, whereas 2 was a monophasic tumor and clustered distinctly. Among the decitabine-treated samples, 1 and 2 were both difficult to group into histological subtypes because they clearly shrank during treatment, leaving little evaluable tumor volume on representative histology sections. Decitabine-treated tumors 3 and 4 were biphasic and poorly differentiated, similar to the 2 vehicle-treated tumors. Of the 5-AZA–treated samples, 1 and 2 were low-density monophasic spindle cell tumors based on the histology sections available. Conversely, 3 and 4 were biphasic/poorly differentiated tumors, similar to the 2 vehicle-treated tumors, 1 and 3. Therefore, we selected for analysis the groups designated as VEH; AZA; and 2 decitabine groups, DAC-A, and DAC-B (Figure 6A). In the RNA-Seq results, DAC-A tumors upregulated 221 genes and downregulated 383 genes relative to VEH tumors, highlighting many immune-related biological processes by GO analysis (Figure 6B). DAC-B treatment upregulated 1,011 genes and downregulated 2,060 genes, with extracellular matrix organization and skeletal system development featuring prominently in GO analysis (Figure 6C). AZA tumors upregulated 490 genes and downregulated 557 genes, featuring mostly skeletal development and extracellular matrix organization, but also some immune-related GO biological processes (Figure 6D). There were few shared genes between the DAC-A and AZA groups, 29 upregulated genes and 101 downregulated genes (Figure 6E). We performed GO analysis focusing on the upregulated groups in the Venn diagrams (Supplemental Figure 5A and 5B). The GO analysis of the 29 genes in common included 4 genes involved in antigen presentation, 3 involved in response to viral infection, and another 3 involved in T cell–related immunity, indicating the induction of an immune response overall (Supplemental Figure 5C). The DAC-B and AZA groups shared 170 genes as commonly upregulated and 446 as commonly downregulated genes (Figure 6F). Similarly, we conducted an analysis focusing on groups with increased expression and found that the AZA-only upregulated genes were enriched for extracellular matrix organization (Supplemental Figure 5D). The genes shared between the AZA and DAC groups were enriched for synapse assembly (Supplemental Figure 5E). The DAC-only upregulated genes were enriched for genes related to the skeletal system (Supplemental Figure 5F).

### Decitabine and 5-AZA alter SS18::SSX distribution.

**Figure 7:**
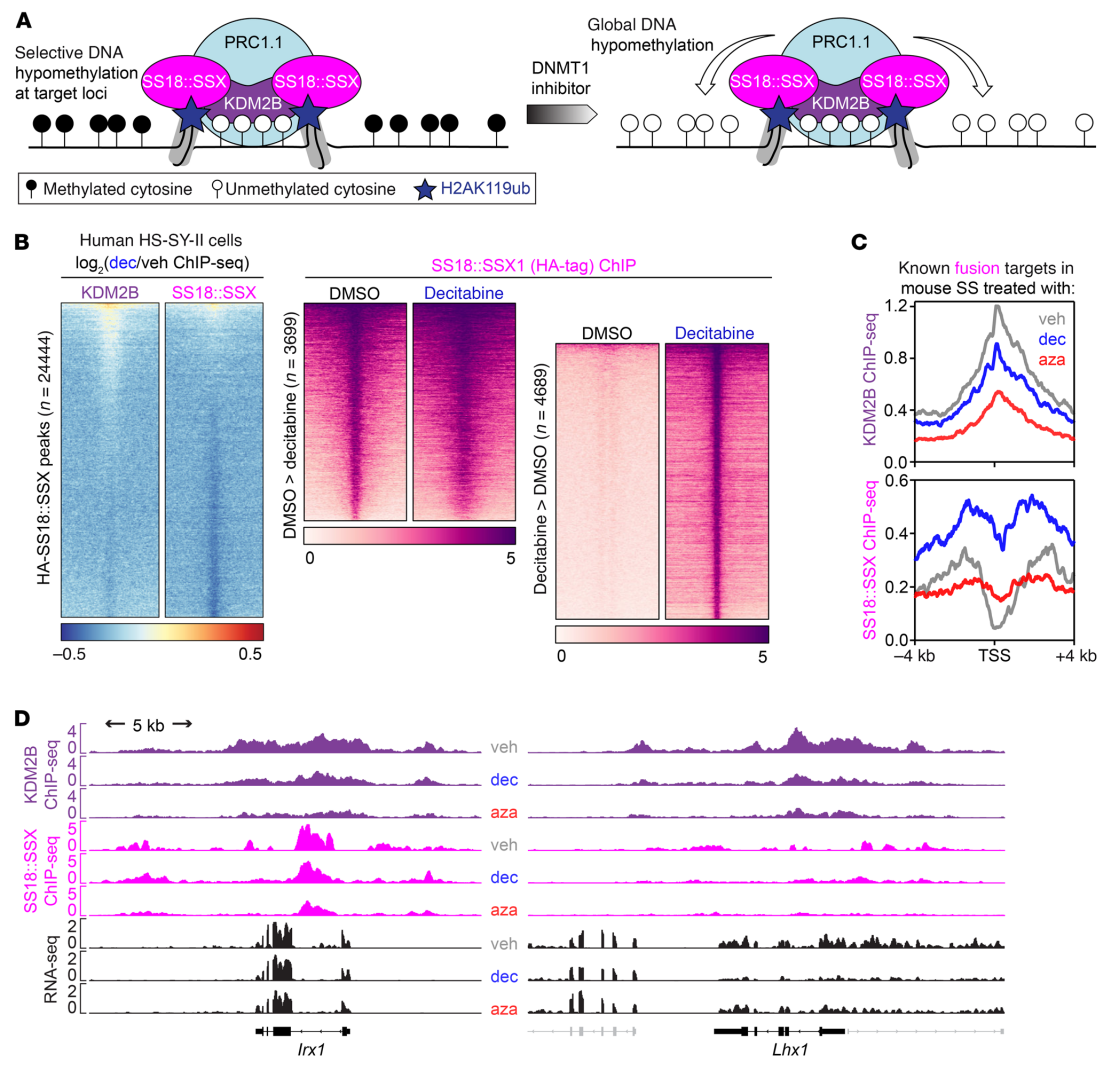
KDM2B redistributes upon DNMT inhibition in synovial sarcoma. (**A**) Schematic diagram illustrating the possibility of SS18::SSX redistribution with increased regions of hypomethylation after DNMT inhibitor treatment. (**B**) Left, heatmaps of the log_2_-transformed fold change KDM2B or SS18-SSX (endogenously HA tagged) calibrated ChIP signals in HS-SY-II cells treated with DMSO or 500 nM decitabine over the total SS18-SSX peaks (DMSO and decitabine, *n* = 24,444). Rows correspond to ±10 kb regions across the midpoint of each enriched region, ranked by increasing signal. Right, heatmaps for SS18::SSX1 calibrated ChIP-Seq over decreased SS18-SSX peaks (*n* = 3,699) or gained peaks (*n* = 4,689). Rows correspond to ±10 kb regions across the midpoint of each enriched region, ranked by increasing signal. (**C**) Enrichment plots for mouse tumor ChIP-Seq for KDM2B and SS18::SSX across the transcription start sites of previously confirmed target genes of the fusion after vehicle, decitabine, or 5-AZA treatment. (**D**) ChIP-Seq track at 2 (rare) example target genes that showed the pattern of diminished KDM2B and fusion ChIP-Seq, as well as RNA-Seq.

Given that SS18::SSX distributes according to the positioning of H2AK119ub, placed by the PRC1.1 complex, which is localized across the genome by KDM2B binding to unmethylated CpG islands, we investigated whether drug-induced changes in DNA methylation affect KDM2B genome occupancy as well as SS18::SSX chromatin binding (Figure 7A). Using ChIP-Seq, we observed regions where decitabine treatment led to the acquisition of new or increased binding sites for SS18::SSX and KDM2B in HS-SY-II human synovial sarcoma cells that did not correspond to previous KDM2B sites (Figure 7B). Decitabine treatment led to a subtle diminution of SS18::SSX presence at a small subset of key regulatory elements, with subsequent rewiring and increased binding at new regions.

A similar phenomenon was clear from ChIP-Seq for KDM2B in mouse synovial sarcomas after treatment with vehicle or decitabine or 5-AZA, with a diminution of ChIP-Seq enrichment at the promoters of known fusion target genes. The diminution of SS18::SSX itself was less consistent (Figure 7C). Only 2 examples of fusion target genes were identified that exhibited diminished KDM2B, SS18::SSX, and RNA-Seq–determined expression (Figure 7D). Such patterns were not consistent across the bulk of fusion target genes, especially for their transcription, which was often increased, in spite of decreased fusion and KDM2B ChIP-Seq enrichment. There are multiple potential explanations for this inconsistency. First, it should be noted that even in tightly controlled cell lines, the diminution of fusion at typical target loci was subtle (Figure 7B). In whole tumor tissues, with intrinsic variability, this effect was even more difficult to detect with confidence. Second, the application of ChIP-Seq to whole tumor samples from the mice tests as much the replacement of necrotic tumor cells with infiltrating fibroblasts and inflammatory cells as it tests the reprogramming of the neoplastic cells’ chromatin. Most importantly, in the mouse tumors and human cell lines, while the redistribution phenomenon markedly affected KDM2B, the target genes of the fusion did not significantly diminish in their transcription (see also Figure 2C, cluster B). There were other gained SS18::SSX binding sites without KDM2B binding that indicated the expression of novel genes from global hypomethylation that recruited canonical BAF complexes in which the fusion was more a passenger than driver of recruitment to those sites. Overall, these results reject the hypothesized mechanism for reduced expression of canonical synovial sarcoma target genes by reduced recruitment of fusion and KDM2B to these loci. Instead, they indicate that because of the hypomethylated CpG islands in synovial sarcoma genomes, these cancer cells are more prone to aberrant gene activation by further global hypomethylation.

### Further genome-wide hypomethylation upregulates gene expression.

**Figure 8:**
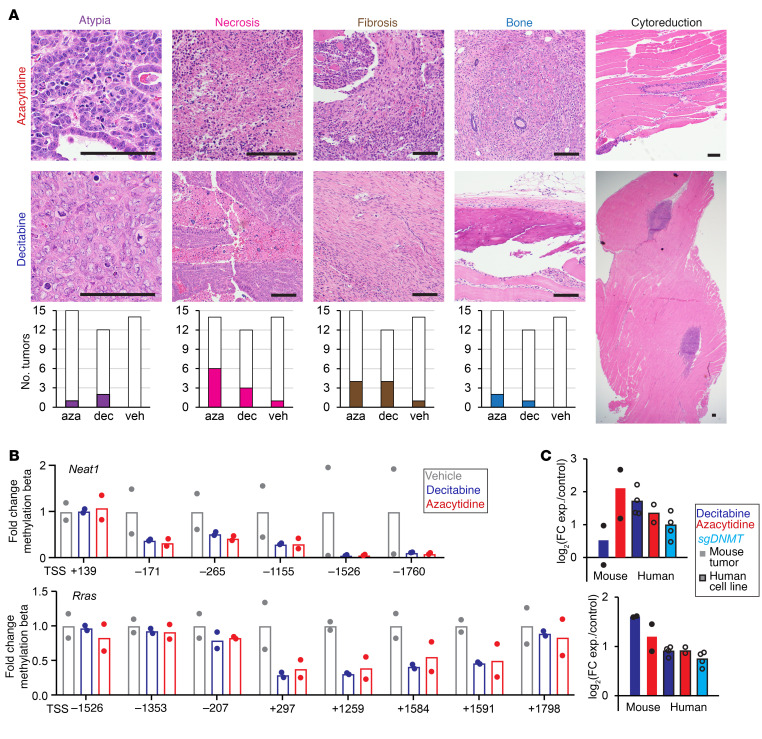
Changes in methylation alter mesenchymal-related and tumor-suppressor genes. (**A**) Example photomicrographs from each experimental group, as well as the prevalence of each finding across all the blindly evaluated samples from each group. Atypia, alterations in nuclear morphology and chromatin characteristics from that seen in normal cells, can be enhanced by DNA damage or cytotoxicity. Necrosis is shown by areas with abundant cells having either pyknotic (dark and small) or absent (ghost cell) nuclei with surrounding neutrophils representing an acute inflammatory response. Fibrosis is the laying down of abundant collagenous extracellular matrix between elongated spindle cells (see Supplemental Figures 3C and 6B for further visualization using Masson’s trichrome). Bone formation in the form of osteoid matrix production with or without mineralization can be observed in some synovial sarcomas, but was only discretely seen in 5-AZA or decitabine samples in this cohort. Cytoreduction was interpreted as present when only very small areas of neoplastic cells were detectable within a background of normal host (skeletal muscle) tissue. (**B**) Promoter methylation fold-changes for genes in vehicle, decitabine-treated, or 5-AZA–treated tumors. (**C**) RNA-Seq–determined log_2_-transformed fold-changes of expression.

Histopathological analysis of the genetically induced synovial sarcomas in mice after treatment with decitabine or 5-AZA revealed the typical treatment effects noted in human tumors after cytotoxic chemotherapy, including increased cytologic and nuclear atypia (characterized by enlarged nuclei, irregular chromatin, and macronucleoli in treated tumor tissues), necrosis, and fibrous replacement of overtly neoplastic tissues (Figure 8A). Masson’s trichrome staining was also performed, and sections from decitabine-treated and 5-AZA–treated mouse tumors stained with abundantly more collagenous matrix than those from vehicle-treated tumors (Supplemental Figure 6, A and B). Some of these changes may be interpreted as conversion of neoplastic cells by derepressed differentiation, consistent with the increased expression of extracellular matrix genes in treated cell lines (Figure 4, C and D). This interpretation of induced/permitted differentiation would better explain the appearance of even osteoid matrix production in extraskeletal regions of genetically initiated tumor treatments because of the frequent observation of osteoid matrix in synovial sarcoma, but less commonly as a treatment effect in most other soft-tissue sarcoma types (36).

We performed integrated analyses of genome-wide methylation arrays, RNA-Seq, and ChIP-Seq to identify epigenetic changes associated with these therapeutic responses. Genes expressed at baseline that were not direct fusion targets and that were further hypomethylated by the 2 demethylase drugs were identified. We evaluated the DAC-B and 5-AZA groups, which were characterized by extracellular matrix organization and skeletal system development in the RNA-Seq GO analysis. The *NEAT1* and *RRAS* genes, which have been reported as tumor suppressor genes (37, 38) or positive regulators of angiogenesis (39) and skeletal muscle tissue development (40) showed substantial decreases in promoter methylation with either treatment (Figure 8B). RNA expression increased in both mouse tumors and human cell lines after drug administration or *DNMT1* disruption (Figure 8C). Focusing on genes related to the extracellular matrix, FLNB is a key factor involved in mesenchymal differentiation and extracellular matrix production. It is part of the cytoskeleton and also has documented gene regulatory functions, especially with regard to alternative splicing of RNA (41, 42). *FLNB* expression increased in conjunction with the decrease in methylation around the promoter (Supplemental Figure 6, C and D). In ChIP-Seq, the SS18::SSX and KDM2B binding regions around the promoter were decreased, suggesting that the increased transcription of this gene was not by a fusion-directed mechanism (Supplemental Figure 6E). In addition, in the drug administration groups, exon skipping was observed to have increased in 2 cell lines upon treatment with either 5-AZA or decitabine, and the expression of *FLNB* may be one reason for the effect on alternative splicing (Supplemental Figure 6F).

**Figure 9:**
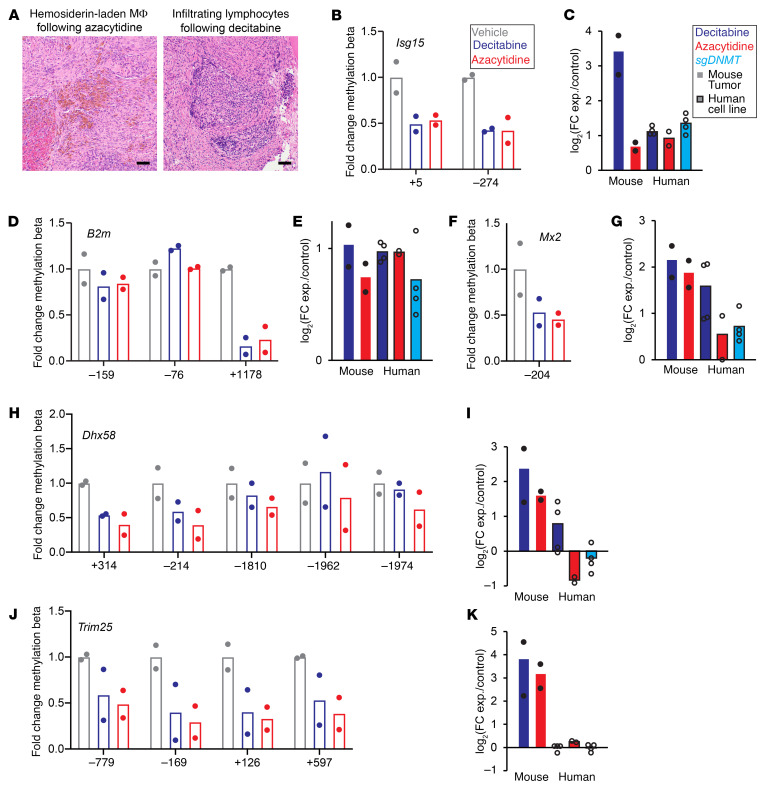
Changes in methylation alter immune-related genes. (**A**) Photomicrographs of H&E-stained sections with abundant hemosiderin-laden macrophages (MΦ) or infiltrating lymphocytes after demethylation therapies in mouse genetically induced tumors. Scale bar: 50 μm. (**B**, **D**, **F**, **H**, and **J**) Promoter methylation fold-changes for noted immune-related genes in vehicle, decitabine-treated, or 5-AZA–treated tumors with matched RNA-Seq–determined log_2_-transformed fold-changes of expression (**C**, **E**, **G**, **I**, and **K**).

We also noted histology evidence of immune-related activity in the treated tumors, ranging from hemosiderin-laden macrophages to infiltrating or clustered lymphocytes in or near the tumors (Figure 9A). We further evaluated the DAC-A and 5-AZA groups for promoter methylation and expression of immune-related genes identified in the RNA-Seq GO analysis. *ISG15, B2M*, and *MX2,* which are related to immunity (43–45), showed substantial decreases in promoter methylation and increases in RNA expression (Figure 9, B–K).

## Discussion

A primary goal in the study of any cancer cell is the identification of biology that is unique to the cancer and distinct from the host’s normal cells. Targeting cancer cell–specific biology with therapy will have a wide therapeutic window, where dosing of the agent can poison the cancer cell without harming the host. The biology of cancer cells provides a variety of categories of such targets, wherein it departs from normal cell biology in meaningful ways. In some instances, what is unique in the cancer cell is the presence or activity of some biology not shared by host cells. In other instances, it is the absence or inactivity of some biology in the cancer cells that host cells typically have present or active. In adolescent and young adult malignancies driven by fusion oncoproteins, current therapeutic strategies have not targeted the unique fusion-associated biology as much as they have targeted pathways that are overrepresented or underrepresented in the cancer cells. Determining which biology is the better target, that which is overrepresented or underrepresented, is a philosophical challenge. Fortuitously, some of the epigenetic mechanisms that determine the reprogramming of gene expression in fusion oncoprotein–associated malignancies have proven to be vulnerabilities when pushed in either direction. This fine-tuning of such biological determinants has been termed the “Goldilocks principle” as related to expression levels of EWSR1::FLI1 in Ewing sarcoma, for example (46).

Here and in other related work, we have characterized profound genome hypomethylation as a unique biology that distinguishes synovial sarcoma from other sarcomas and most healthy cells of a host (29). Such hypomethylation might derive from overactive demethylating enzymes or underactive methylating enzymes. One could propose to target either of these therapeutically. In synovial sarcoma tumors, we found increased expression of demethylating enzymes and their targeting for upregulated transcription by the fusion oncoprotein (29). However, single gene dependency testing identified only *DNMT1* among those 2 gene groups as a particular point of vulnerability, even if the observed state of synovial sarcoma suggested relatively low activity of this enzyme in the cancer cells.

We confirmed that application of purported DNMT1 inhibitors in synovial sarcoma cell lines strongly recapitulated the impact of *DNMT1* gene disruption in transcription and cell morphology in tissue culture. We also identified that such small molecule inhibitors are effective at suppressing growth of cell lines in culture or in a xenograft context. More strikingly, the drugs provided not only substantial growth suppression, but even cytoreduction in mouse genetically induced synovial sarcomas. The persistent growth suppression that lasted for weeks after a brief period of drug application appears particularly promising for clinical translation of these agents to patients with synovial sarcoma.

High expression of DNMT1 is associated with poor breast cancer survival and progression of disease (47–49). In many of the hematological malignancies for which DNMT1 inhibitors are used clinically, there is also elevated expression of the protein. This is not the case in synovial sarcoma, making its DNMT1 vulnerability somehow related instead to reduced baseline activity.

In our efforts to understand the mechanism of this sensitivity of synovial sarcoma to DNMT1 inhibitors, we tested a few potential mechanisms. Although the unique biology of fusion oncoprotein distribution across the chromatin of the genome depends on the hypomethylated state of developmental gene promoters, and further hypomethylation of other loci drove a redistribution effect on the fusion and reduction of its presence at typically targeted gene regulatory elements, this only rarely resulted in reduced expression of these target genes. Depletion of SS18::SSX only shared transcriptional downregulation with DNMT1 disruption or inhibition at a few genes. Instead, DNMT1 inhibition or disruption matched SS18::SSX depletion in a subset of the genes that were upregulated from fusion depletion. The increased expression of non-fusion–targeted genes that were relatively suppressed transcriptionally at baseline in synovial sarcoma cells had a more prominent impact on the application of *DNMT1* silencing, which included genes that drove mesenchymal differentiation and extracellular matrix production, as well as tumor suppressor genes.

Further, although the epigenetic reprogramming characteristic of synovial sarcoma development already derepresses expression of several cancer-testis antigens (50, 51), the expression of these antigens may be even more pronounced when demethylating agents are applied therapeutically. This likely explains some of the increased efficacy noted for these agents in our immune-competent mouse genetic models over the xenografts of a human cell line.

## Methods

### Sex as a biological variable.

For all mouse genetic model experiments, littermate controls were used, including randomly assigned male and female mice to each group. For the SYO-1 xenograft group, only female mice were used because that cell line was derived from a tumor procured from a female patient. No sex-based differences were grossly observed in these experiments.

### Cell lines.

HS-SY-II, SYO-1, ASKA, YaFuSS, and 1273/99 cell lines were provided by provided by M. Ladanyi (Memorial Sloan-Kettering Cancer Center, New York, New York, USA) and Priya Chudasama (German Cancer Research Center, Heidelberg, Germany) (52–56). MoJo was generated in the laboratory (57). Human osteosarcoma KHOS-240S (RRID:CVCL_2544), HEK293T (RRID:CVCL_0063), and immortalized human endometrial stromal cells KC02-44D hTERT (RRID:CVCL_E224) were purchased from ATCC. The cell line 1273/99 was cultured in RPMI 1640 (Gibco); other cell lines were cultured in DMEM (Gibco) supplemented with 10% FBS (Thermo Fisher Scientific) and 100 IU/mL penicillin/streptomycin (Gibco) and maintained in a humidified incubator at 37°C with 5% CO_2_ (52). hTERT immortalized adipose-derived mesenchymal stem cells were purchased from ATCC (SCRC-4000) and were cultured in MesenPRO RS medium (Gibco, 12746-012) supplemented with L-glutamine (Sigma-Aldrich, G7513-100ML). RAG MEF cells were procured in-house (Memorial Sloan Kettering Cancer Center [MSKCC]) and maintained in DMEM media supplemented with 10% FBS. Cells treated with decitabine (Sigma-Aldrich; stock 10 mM in DMSO) had the media changed every 48 hours for 6 days (growth assay) or 12 days (Dot Blot, RNA-Seq, ChIP-Seq).

### Virus production and transduction.

Lentiviruses were produced by cotransfection of HEK293T cells. For a 6-well plate, 1 × 10^6^ cells were plated the day before transfection with 3 g constructs and helper vectors (2.5 g psPAX2 and 0.9 g VSV-G). Transfection of packaging cells was performed using polyethylenimine (Polysciences, 23966-2) by mixing with DNA in a 3:1 ratio. Viral supernatants were collected 48 hours after transfection, filtered through a 0.45 m filter, and supplemented with 4 g/mL of polybrene (Sigma-Aldrich) before adding to target cells. Downstream experiments using DNMT1 sgRNA knockout were performed 12 days after sgRNA induction.

### Stable Cas9-expressing cell generation.

The HS-SY-II synovial sarcoma cell line was transduced with lentiCas9-Blast (58) (Addgene plasmid 52962) and selected using 5 g/mL of blasticidin to generate stable Cas9-expressing cell lines. Cells were consequently transduced with DNMT1 sgRNAs and selected with puromycin.

### Plasmid cloning.

DNMT1 sgRNAs for CRISPR knockout were designed using Sanjana lab tool (http://sanjanalab.org/home.html) and cloned using their previously described protocol (34, 58). Briefly, sgRNAs were cloned by annealing 2 DNA oligos and ligating into a BsmB1-digested pLKO1-puro-U6-sgRNA-eGFP. Transformation was carried into Stbl3 bacteria. The sequences are sgCTRL: CACCGTCCCATTCCTGGCCATTCT; sgDNMT1 1: CACCGCAGTCCTCTGTGAACACTG; sgDNMT1 2 CACCGCATCGAGATGTGGGACCCT.

### Western blotting.

Cells grown in a 6-well plate were harvested and washed in PBS. Cell pellets were incubated with RIPA buffer (Cell Signaling Technology) supplemented with protease inhibitor tablets (Roche) for 30 minutes and cleared by centrifugation (15 minutes; 14,000 x g at 4°C). Protein lysates were quantified using the BCA protein assay (Pierce). Lysates were then denatured in 2× Laemmli (Thermo Fisher Scientific) at 95°C for 10 minutes and run in Mini-PROTEAN Precast gels (Bio-Rad) and transferred into membranes using Trans-Blot Turbo. Membranes were blocked in 5% milk. The following antibodies were used for immunoblotting: β-actin HRP clone AC-15 (A3854, Sigma-Aldrich), DNMT1 (D63A6) XP (5032, Cell Signaling Technology). Western blots were visualized using Amersham Imager 680.

### Dot blot.

Genomic DNA from a confluent 6-well plate was isolated using DNeasy Blood & Tissue kits (QIAGEN) according to the manufacturer’s protocol. DNA was resuspended in 50 μL TE buffer. Next, 1 μg and 500 ng DNA samples were added to up to 10 μL or 20 μL of denaturation buffer (0.4 mM NaOH, 10 mM EDTA). DNA was denatured at 100°C for 10 minutes before being cooled on ice for 5 minutes. 2.5 μL samples were then applied to a positively charged nylon membrane under vacuum using a 96-well Dot Blot Hybridization Manifold (Harvard Apparatus Limited) and left to dry for 15 minutes. Membrane was then UV-cross-linked at 150 mJ/cm^2^ and washed in 2× SSC. A second cross-link was applied, and membranes were left to dry for 6 hours at room temperature. Membranes were then blocked in 10% milk and 1% BSA in PBT (PBS + 0.1% Tween 20) overnight at 4°C. Hybond N+ membranes were probed with antibodies specific to OptimAb anti-5-methylcytosine (33D3) (BI-MECY-0100, Eurogentec, dilution factor 1:1000) for 1 hour at room temperature followed by Mouse IgG HRP Linked Whole Ab (NXA931, Sigma-Aldrich,1:10000) for 1 hour at room temperature. Membranes were then enhanced with ECL and visualized using Amersham Imager 680. Methylene blue staining was carried out after this as loading control.

### Cell competition assays.

HS-SY-II and KHOS-240S Cas9 cells were transduced with an empty plasmid (empty vector) or a plasmid containing sgRNA targeting *DNMT1*. Infections were done with a virus dilution of 1:10 to obtain an infection efficiency of around 70%–80%. Infected cells became GFP+ because of the backbone of the sgRNA. The cells were then cultured over a period of 25 days, and the percentage of GFP+ cells was measured using a Fortessa FACS machine. Data were analyzed using FlowJo software (Becton, Dickinson & Company).

### RNA extraction and real-time qPCR.

RNA was prepared using RNeasy mini kit (QIAGEN) according to the manufacturer’s protocol, including a DNaseI (QIAGEN) treatment for 15 minutes at room temperature; cDNA was obtained using RevertAid First Strand cDNA Synthesis kit (Thermo Fisher Scientific).

Real-time PCR was carried on the Roche LightCycler 480 Real-Time PCR System using SYBR Green PCR Master Mix (Applied Biosystems). The relative expression of each sample was measured by the LightCycler software and normalized to the mean for *Gapdh* from replicates. Finally, the log_2_ of the ratio relative to DMSO-treated cells or safe guide induced cells was calculated when mentioned. Expression primers were the following: BCAS1(F), TTCTTCTGGTGTCCTGTGGA; BCAS1(R), TGGTAAGTCTCTGCTTCTGGT; DAPK1(F), GCAGGAAAACGTGGATGATT; DAPK1(R), CATTTCTTCACAACCGCAAA; DNAH3(F), GAACAACCTCCAGACGCG; DNAH3(R), TGGAAATGATATTCAGATGGCGA; DNAH12(F), CTCCCAACACATCCGACCAT; DNAH12(R), TTTTCCTGTGCCTGCTGG; ERC1(F), TGATTTTCTCTTGCTGCCGC; ERC1(R), CGCAATTCATCCTGGAGAGC.

### RNA-Seq and data analysis of HS-SY-II cell line with CRISPR and decitabine.

For high-throughput RNA-Seq, total RNA from 2 independent experiments was extracted using a RNeasy mini-kit (QIAGEN). Cells transduced with the indicated sgRNAs or treated with decitabine were collected 12 days after infection. RNA-Seq library preparation and sequencing were performed at the High Throughput Sequencing Unit of the Genomics & Proteomics core facility of DKFZ using TruSeq Stranded Total RNA Library Prep kit (Illumina) and HiSeq 2000 v4 single-read 50 bp sequencing system.

RNA-Seq reads were aligned to the human genome assembly hg19, and the FPKM count matrix was generated using featureCounts (59). Data analysis of replicate clustering (principal component analysis), heatmaps of the 5,000 most variable genes, and differential expression analysis were performed using iDEP (http://bioinformatics.sdstate.edu/idep93/) (60). A sample-to-sample correlation heatmap was generated using DESeq2 (61).

### RNA-Seq and data analysis of mouse model tumors and human cell lines.

RNA was extracted from samples using TRIzol (Thermo Fisher Scientific,15596018), followed by chloroform to 16.67% by volume. RNA was then purified from the suspended lysate and eluted in water using a kit (Zymo Research, RNA Clean & Concentrator R1018). RNA-Seq libraries were prepared using a NEBNext Ultra II Directional RNA Library Prep with rRNA Depletion kit (human, mouse, rat) (NEB) and sequenced on an IlluminaX Series 10B Reagent kit (150 × 150 bp sequencing; 1,250 M read-pairs/lane). The quality of raw paired-end sequence reads was assessed using FastQC. Low-quality (<20) bases and adapter sequences were trimmed using Trimmomatic software (version 0.38) with the following parameters: ILLUMINACLIP: path/to/adapter.fa:2:30:10 LEADING:20 TRAILING:20 SLIDINGWINDOW:4:15 MINLEN:36. The trimmed reads were aligned to the reference genome (mouse genome version mm10 and human genome version hg38) using the HISAT2 RNA-Seq aligner (version 2.1.0). The raw read counts were normalized by relative log normalization, and differential expression analysis was conducted with DESeq2 (version 1.24.0). Differentially expressed genes were detected with thresholds of |log_2_FC (fold change)| greater than 1 and adjusted *P* value less than 0.05 using the Benjamini-Hochberg method. GO enrichment analysis of differentially expressed genes was performed using GOATOOLS (version 1.1.6). Venn diagrams were plotted using the R package ggVennDiagram (version 1.2.0). Variance analysis was performed using the TPM values of the RNA-Seq data. Additionally, k-means clustering analysis was performed on the relevant genes identified using variance analysis. A heatmap was plotted based on the k-means clustering analysis results.

### Robust and flexible detection of differential alternative splicing from replicate (rMATS).

FASTQ files obtained from RNA-Seq of HS-SY-II and SYO-1 cells treated with 5-AZA or decitabine were aligned to the reference genome, and splicing event quantification was performed using rMATS Turbo (version 4.1.2) (62). In this analysis, we used the junction counts and exon coverage mode to ensure comprehensive event detection, incorporating both exon inclusion and skipping events in the overall count estimation. The percentage spliced-in (PSI, Ψ) metric was used to measure the relative abundance of an exon in the spliced isoform. Statistically significant exon skipping events were identified based on an FDR threshold of less than 0.05, which was calculated using the Benjamini-Hochberg correction to control for multiple testing.

### Immunofluorescence and image capture.

HS-SY-II or SYO-I cells were seeded in 6-well plates at a density of 200,000 cells per well and treated for 9 days with 500 nM decitabine or a corresponding amount of DMSO for the control. On day 9, the cells were resuspended and seeded onto glass cover slips to allow for proper adherence. Treatment was performed for a total of 12 days. The cells were fixed with 4% PFA for 10 minutes at room temperature, permeabilized with 0.2% Triton X-100 in PBS for12 minutes at room temperature, and blocked with immunofluorescence blocking solution (0.5% BSA and 0.2% fish skin gelatin in PBS) for 1 hour at room temperature. Incubation with the mouse β-actin (ac-15, Sigma-Aldrich, 1:600) antibody was performed in blocking buffer for 1 hour at room temperature, followed by incubation with anti-mouse-Alexa Fluor 488 secondary antibody (A11001, Invitrogen, Thermo Fisher Scientific, 1:400) for 1 hour at room temperature. Alternatively, phalloidin-Alexa Fluor 647 (A22287, Invitrogen, Thermo Fisher Scientific) was diluted at 1:700 ratio in blocking solution, and the samples were incubated with it for 1 hour at room temperature, followed by 3 washes with PBS. The slides were mounted in Vectashield antifade mounting medium with DAPI, sealed with clear nail polish, and imaged on an SP8 Leica confocal laser scanning microscope using a 40× oil immersion objective, 1024 × 1024 pixel image size, 100 Hz. Image analysis was performed using Fiji (ImageJ [NIH]).

### In vitro drug treatment.

Cells were treated for 6 days and the drugs refreshed after 3 days. The viability assay was measured with Cell Titer Glow. Cells were seeded in duplicate at different numbers, allowing growth for 6 days.

### Calibrated cross-linked ChIP in human cell lines.

A modified ChIP protocol was used to analyze SS18::SSX1 binding in synovial sarcoma cells. HSSY-II cells with endogenously HA-tagged SS18::SSX and murine RAG-MEF cells overexpressing HA-SS18::SSX were cultured, with the latter used for spike-in controls. After 12 days of 500 nM decitabine or DMSO treatment in triplicate, 10 million HSSY-II were harvested and fixed using EGS (Thermo Fisher Scientific, 21565) and 1% formaldehyde (Thermo Fisher Scientific, 28908). Nuclei were isolated and sonicated using a Covaris ultrasonicator (M220). Each sample was sheared for 30 minutes under the following conditions: duty factor = 10%, peak power = 75.0, cycles/burst = 200. Sheared chromatin was incubated with 5 μg HA-tag (C29F4) rabbit mAb (3724, Cell Signaling Technology) per sample overnight at 4°C on a rotator, followed by IP using magnetic beads. After washing, DNA was purified using the QIAGEN PCR purification kit (28104).

### Library preparation of human cell lines using calibrated cross-linked ChIP.

DNA fragments obtained after ChIP were quantified using Qubit dsDNA HS Assay kit (Invitrogen). Next, 5 ng of DNA was used for library preparation using NEBNext Ultra II DNA Library Prep kit for Illumina (NEB, E7645S), SPRIselect beads (Beckman Coulter, B23317), and NEBNext Multiplex Oligos for Illumina (NEB, Set 1 E7335S) following NEB’s guidelines (NEB, E7645S). All the libraries were done without size selection with an input of 5 ng. ChIP libraries were sequenced as 50 bp paired-end reads on NovaSeq 6K SP.

### Calibrated cross-linked ChIP-Seq analysis of human cell line data.

Raw reads were trimmed for quality and Illumina adapter sequences using Trim Galore, then aligned to the human genome assembly T2T-CHM13 using Bowtie 2 (with “--very-sensitive” option). The spike-in data were analyzed by alignment with mouse genome mm10. The reads from the spike-in were used to normalize the reads. PCR duplicates were removed using Rmdup tool. Down-sampling of reads for each sample was done based on the formula from Fursova et al. (Equation 1)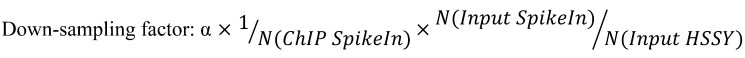:

where α is a coefficient applied for all the files normalized together so the value of the largest down-sampling factor equals 1. N(ChIP SpikeIn) is the total number of reads aligned to the dm6 in the IP sample; N(Input SpikeIn) is the total number of reads aligned to mm10 in the corresponding Input; N(Input HSSY) is the total number of reads aligned to the T2T genome in the corresponding Input sample. The down-sampled replicates were then combined using the pileup function from MACS2 (*q* value 0.05), and bigWig files were generated with the ucsc-wigtobigwig tool. To visualize ChIP-Seq tracks, normalized bigWig files were generated with the ucsc-wigtobigwig tool. Peaks were generated with the MACS2 call peak function (with “--no model”, “--qvalue 0.01”, “--broad“ options). CpG islands were imported from the UCSC genome browser (https://genome.ucsc.edu/).

### Calibrated cross-linked ChIP data visualization of human cell line data.

Genome tracks were visualized using the UCSC genome browser. For heatmaps and metaplot profiles, read densities of the various IPs were centered around peak signals with a +/– 5 kb window from peak center and binned with 50 bp using computeMatrix and plotProfile/plotHeatmap functions from deepTools.

### Mouse genetic model of synovial sarcoma.

The *Rosa26^hSS2^* and *Myf5-Cre* mouse strains were previously generated in our laboratory (9). Mouse tumor growth progression was measured using calipers and calculated using an ellipsoidal formula: volume = (length × width × width) × 0.5. Mice were monitored for signs of distress that indicated euthanasia via CO_2,_ followed by cervical dislocation. Tumors were surgically removed using sterilized scalpels and forceps, placed into 1.5 mL tubes, and immediately frozen in liquid N_2_. The experiment included both male and female mice, with no observed differences between them in the effects of drug treatments.

### ChIP-Seq of mouse model tumors.

Snap-frozen tumor specimens were pulverized to near single-cell size with a hammer in the frozen state. For routine ChIP, pulverized tumors were thawed in PBS containing 1% formaldehyde and quenched with glycine (125 mM) after 10 minutes. Cells were pelleted and washed 3 times with cold PBS. Nuclei were isolated by dousing in a solution of Tris-HCl (10 mM, pH 8.5), NaCl (10 mM), and NP-40 (0.5% volume) with protease inhibitor (PI, Sigma-Aldrich). They were then washed in a solution of Tris-HCl (10 mM, pH 8.5), NaCl (200 mM), EDTA (1 mM), and 1% SDS with PI. Nuclei were lysed at 4°C in a solution of Tris-HCl (50 mM, pH 8.0), EDTA (10 mM), and 1% SDS with PI and immediately diluted 1:10 with a solution of 16.7 mM Tris-HCl pH 8.1, 16.7 mM NaCl, 1.2 mM EDTA, 1.1% Triton X-100, and 0.01% SDS with PI. Samples were sonicated with an EpiShear probe sonicator (Active Motif) at 40% amplitude for 8 cycles of 30 seconds each. Precipitated nuclear components that were not solubilized by sonication were centrifuged and removed. Dynabeads-Rabbit (30 μL; Thermo Fisher Scientific) were washed 3 times in PBS with BSA (0.5 mg/mL), added to the sonicated chromatin, and incubated for 1 hour at 4°C for preclearance. The supernatant was transferred to a new tube. One subsample was set aside as input. Sonicated chromatin was incubated with 5 μg of a primary antibody: anti-SS18::SSX (72364S, Cell Signaling Technology) and KDM2B (17-10264, Sigma-Aldrich) overnight with rotation at 4°C. The next day, 100 μL of the washed Dynabead slurry was incubated with the immunoprecipitated chromatin for 4.5 hours. The beads were then collected on a magnetic stand and washed sequentially with 6× ChIP RIPA buffer (10 mM Tris-HCI pH 7.5, 140 mM NaCl, 1 mM EDTA, 0.5 mM EGTA, 1% NP-40 or Triton X-100, 0. 1% SDS, and 0.1% NaDOC), 2× ChIP LiCl wash buffer (10 mM Tris-HCI pH 8.0, 1 mM EDTA, 500 mM LiCl, 0.5% NP-40, 0.5% NaDOC), and 1× with TE (10 mM Tris-HCI pH 8.0, 1 mM EDTA). Samples were eluted from the beads with buffer containing Tris-HCl (10 mM, pH 8.0), NaCl (150 mM), EDTA (5 mM), and SDS (1%). Cross-linking was reversed by adding and incubating with 40 μg of Proteinase K at 37°C for 60 minutes and 65°C for 3 hours. DNA was purified using a DNA Clean & Concentrator kit (Zymo). DNA was synthesized into sequencing libraries using the ChIP-Seq with NEBNext Ultra II DNA Library Prep kit and then sequenced using NovaSeq X Series 10B Reagent kit (150 × 150 bp sequencing;1,250 M read-pairs/lane).

### ChIP-Seq analysis of mouse model tumors.

Reads were aligned to the mm10 mouse genome version using Novoalign (version 3.00) for paired-end reads. Peaks were called from each of the aligned bam files against input reads using MACS2 (version 2.2.9) (63), with the parameters callpeak -B --SPMR --qvalue = 1e-3 --mfold 15 100. ChIP input was used as the background for MACS2. MACS was used to produce normalized bedgraphs, which were subsequently converted to bigWig files. Peaks were filtered to remove peaks that are in blacklist, including ENCODE blacklisted regions (64). Duplicate reads were removed using samtools rmdup for all downstream analyses (65). Merged bigWig enrichment files for each condition with multiple replicas were generated in an average manner followed by normalization of read depth.

Heatmaps and profile plots that illustrate the scores corresponding to genomic regions were produced using the plotHeatmap function in deepTools (version 3.5.6) (66). This followed the computation of scores for each genomic region, a process that was executed using the computeMatrix function.

### DNA methylation array.

DNA methylation array experiments were performed using the Infinium Mouse Methylation BeadChip (Illumina). Approximately 500 ng of genomic DNA was bisulfite-converted with the Zymo Research EZ DNA Methylation kit (Illumina) with a slight modification. Samples were processed in a thermal cycler under the following conditions: 95°C for 30 seconds, 50°C for 60 minutes for 16 cycles, and held at 4°C. Data were processed using the Infinium Mouse Methylation BeadChip and Illumina Infinium HD MouseMethylation-12 v1.0 arrays. Utilizing SeSAMe software (version 1.20.0), the beta and M values for each sample were obtained after general preprocessing of the IDAT file. Next, Illumina software was used to detect differentially methylated probes (DMP) based on the M value. The probes with hyper (log FC > 1and *P* values < 0.05) or hypo (log FC ≤ 1and *P* values < 0.05) methylated on 5-AZA disposure and those on decitabine were compared, and their common and specific probes were identified.

### GEMM drug treatment.

After tumors had developed to a size greater than 400 mm^3^, animals were randomized into 3 groups, which received i.p. administered vehicle (saline), 5 mg/kg decitabine, or 5 mg/kg 5-AZA on 4 consecutive days per week. Tumor volume was calculated using the following formula: 0.5 × (large diameter) × (small diameter)^2^. The mice were euthanized whenever a tumor’s volume reached greater than 2,000 mm^3^.

### Xenograft experiments.

To generate xenografts, SYO-1 cells (15 × 10^6^ per mouse flank) were harvested and injected in growth factor–reduced Matrigel/PBS (50% final concentration). Each mouse flank was s.c. injected. Female 5- to 7-week-old athymic Nude-*Foxn1^nu^* (Envigo) mice were used. After inoculation, mice were monitored for tumor growth, and caliper measurements started when tumors became visible. Tumor volumes were calculated using the following formula: tumor volume = (D × d2)/2, in which D and d refer to the long and short tumor diameter, respectively. When tumors reached approximately 100 mm^3^, decitabine treatment was initiated using 3 mg/kg i.p., 3 times per week over 2 weeks. SYO-1–derived xenograft tumors were harvested at the final time point and weighted. All mouse experiments were approved by the MSKCC IACUC.

### Tumor processing for histology.

Tumor samples were fixed in 4% formaldehyde, dehydrated in serial ethanol solutions to xylene, embedded in paraffin, sliced into 4 μm sections with a microtome, and mounted on slides. Slides were deparaffinized with xylene, rehydrated with serial ethanol, and stained with H&E or Masson’s trichrome for light microscopy viewing.

### Treatments and assessment of necrosis and apoptosis by flow cytometry.

To quantify necrosis and apoptosis, the CellEvent Caspase-3/7 Green Flow Cytometry Assay kit (Thermo Fisher Scientific) was used. Cells were labeled with CellEvent caspase 3/7 green detection reagent for 25 minutes at 37°C; SYTOX AADvanced dead cell stain solution was added, and the cells were incubated for 5 minutes. A total of 10,0000 stained cells per sample were acquired and analyzed with a flow cytometer using Kaluza software version 2.3 (Beckman Coulter).

### Statistics.

All statistical methods used were 2-tailed and included a baseline level of significance threshold at *P* less than 0.05. Graphs were created and analyzed using GraphPad Prism 10. All quantitative data are presented as mean ± SD. Methods used included the Mann-Whitney *U* test and paired or homoscedastic *t* tests (2 tailed, the latter for presumed equal variance, but not pairing), as noted in the figure legends.

### Study approval.

Experiments with the mouse genetic model of synovial sarcoma were performed with the approval of the University of Utah IACUC and per international law and humane principles. All associated murine protocols were described under protocol 1442, last reapproved in November 2024. The mouse xenograft experiments were approved by the MSKCC IACUC.

### Data availability.

All underlying data used to generate the figures in this study are available in the Supporting Data Values file in the supplemental material. All genomic data have been uploaded to the NCBI’s Gene Expression Omnibus (GEO) under the accession numbers GSE291441, GSE291301, GSE291302, GSE291312, GSE291314, and GSE292622.

## Author contributions

NH, NSB, KSF, SM, JL, CAJ, LW, VD, VG, and AP performed experiments. NH, NSB, and LL analyzed data. LC, MH, SMP, SWL, TON, AB, and KBJ supervised experiments and designed analyses. The paper was written primarily by NH, NSB, TON, AB, and KBJ and edited by all authors.

## Supplementary Material

Supplemental data

Unedited blot and gel images

Supporting data values

## Version 1 04/29/2025

In-Press Preview

## Version 2 07/01/2025

Electronic publication
