# Supplementary material for: DNA demethylating agents suppress preclinical models of synovial sarcoma
Source: J Clin Invest. 2025 Apr 29;135(13):e190855. doi: 10.1172/JCI190855 (PMC12208545; doi:10.1172/JCI190855)
Supplement: Supplemental data [file jci-135-190855-s198.pdf]

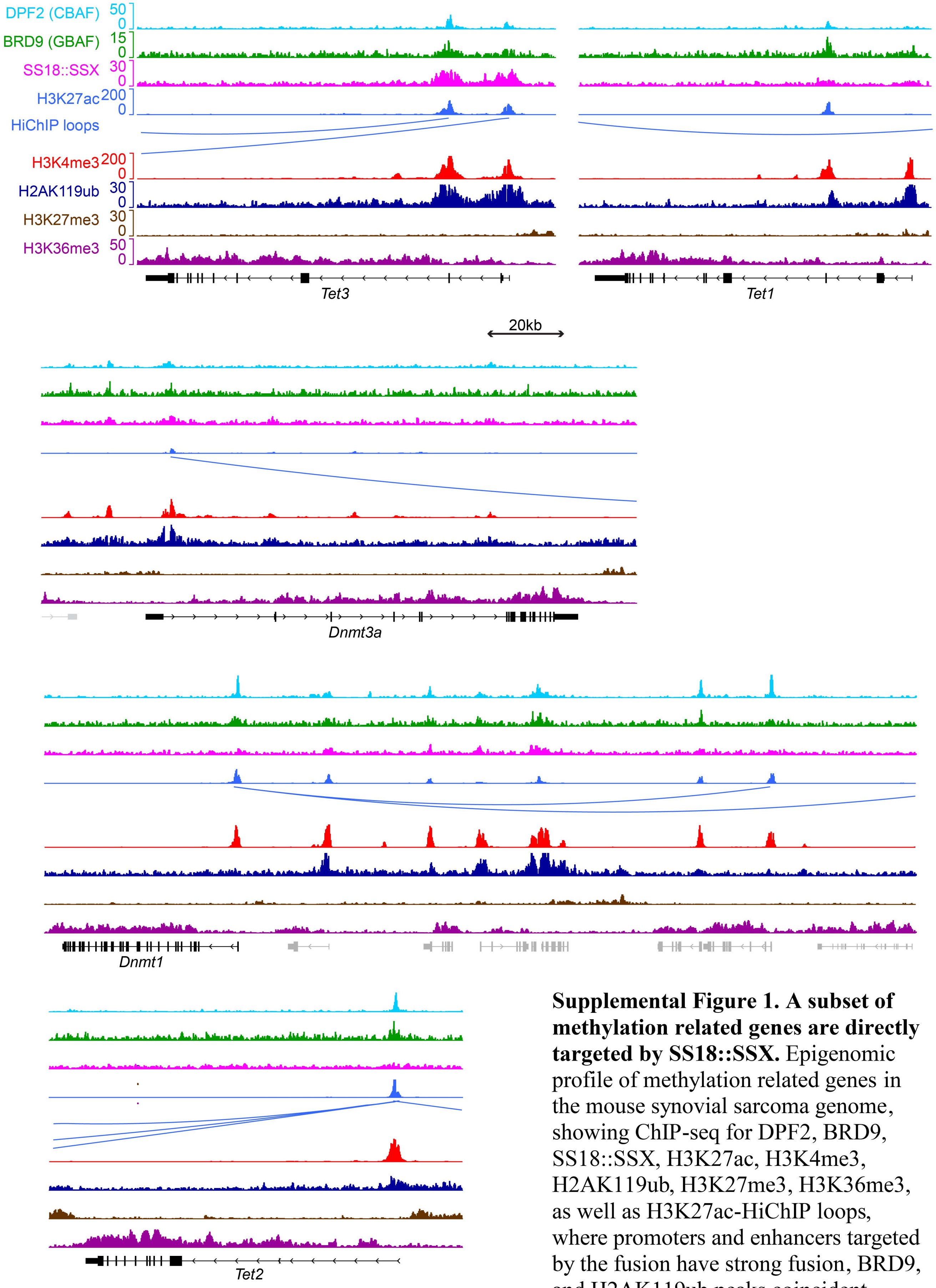


**Supplemental Figure 1. A subset of methylation related genes are directly targeted by SS18::SSX.** Epigenomic profile of methylation related genes in the mouse synovial sarcoma genome, showing ChIP-seq for DPF2, BRD9, SS18::SSX, H3K27ac, H3K4me3, H2AK119ub, H3K27me3, H3K36me3, as well as H3K27ac-HiChIP loops, where promoters and enhancers targeted by the fusion have strong fusion, BRD9, and H2AK119ub peaks coincident.

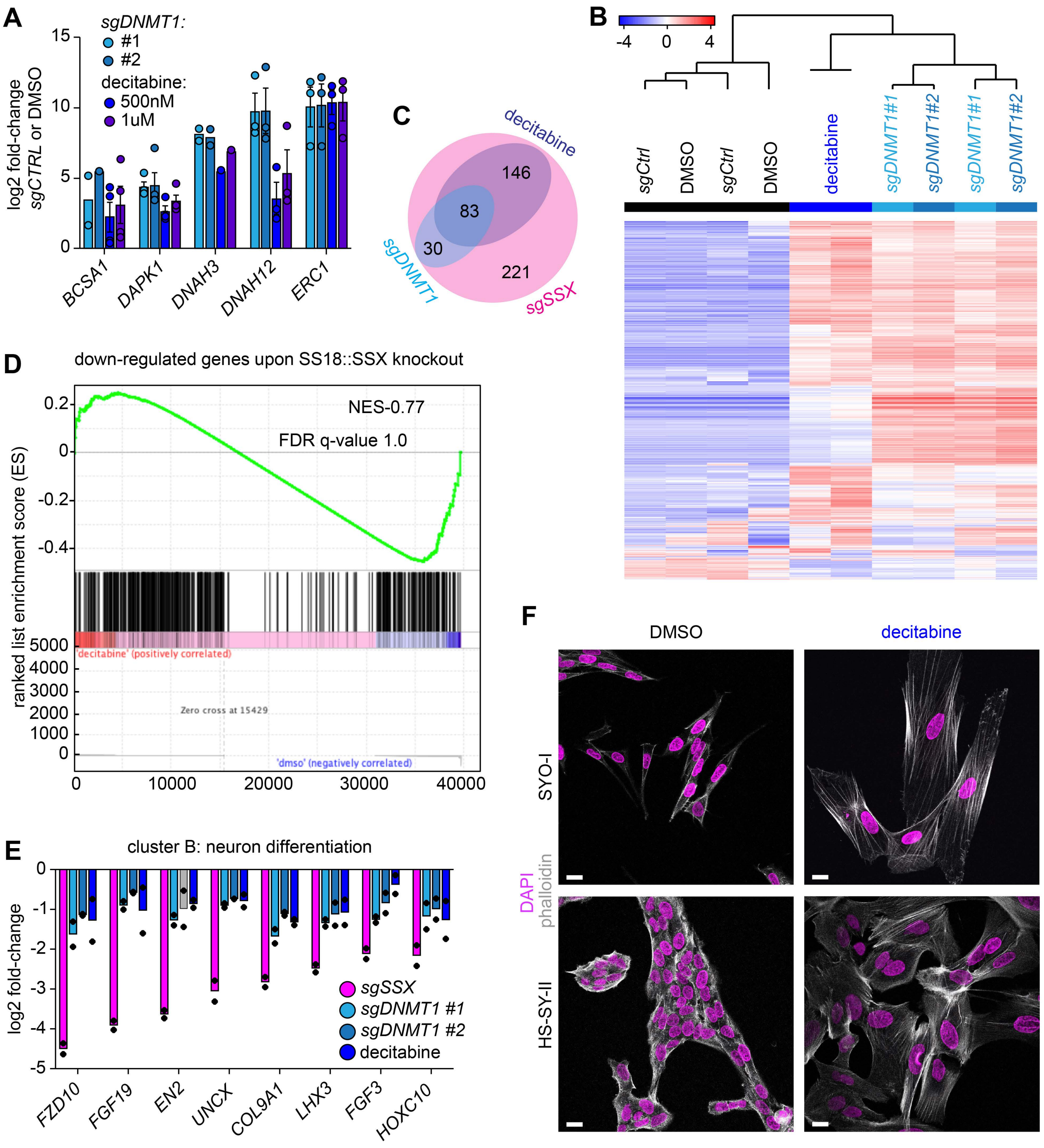


**Supplemental Figure 2. DNMT1 inhibition or knock-out impacts transcription.** (**A**) qRT-PCR of methylation sensitive promoters 12 days after *sgDNMT1* or decitabine treatment. Data represents the mean of n = 1-4 biological replicates. (**B**) RNA-seq heatmap showing 5000 most variable genes with a cut off z score of 4. (**C**) Venn diagram of genes for which log2 fold-changes of FPKM values were greater than 0.5, demonstrating the overlap of three groups from cluster C in Figure 2C. (**D**) Gene set enrichment analysis (GSEA) comparing the expression of the 411 genes present in cluster B in Figure 2C, neuron differentiation, with the top 500 genes downregulated upon SS18::SSX knockout. (**E**) Log2 fold-change of FPKM values from genes present in cluster B. Data represent the mean of two biological replicates. (**F**) Immunofluorescence of human SS HS-SY-II and SYO-1 cells stained with DAPI (magenta) and phalloidin (grey). Scale bars are 20µm in length.

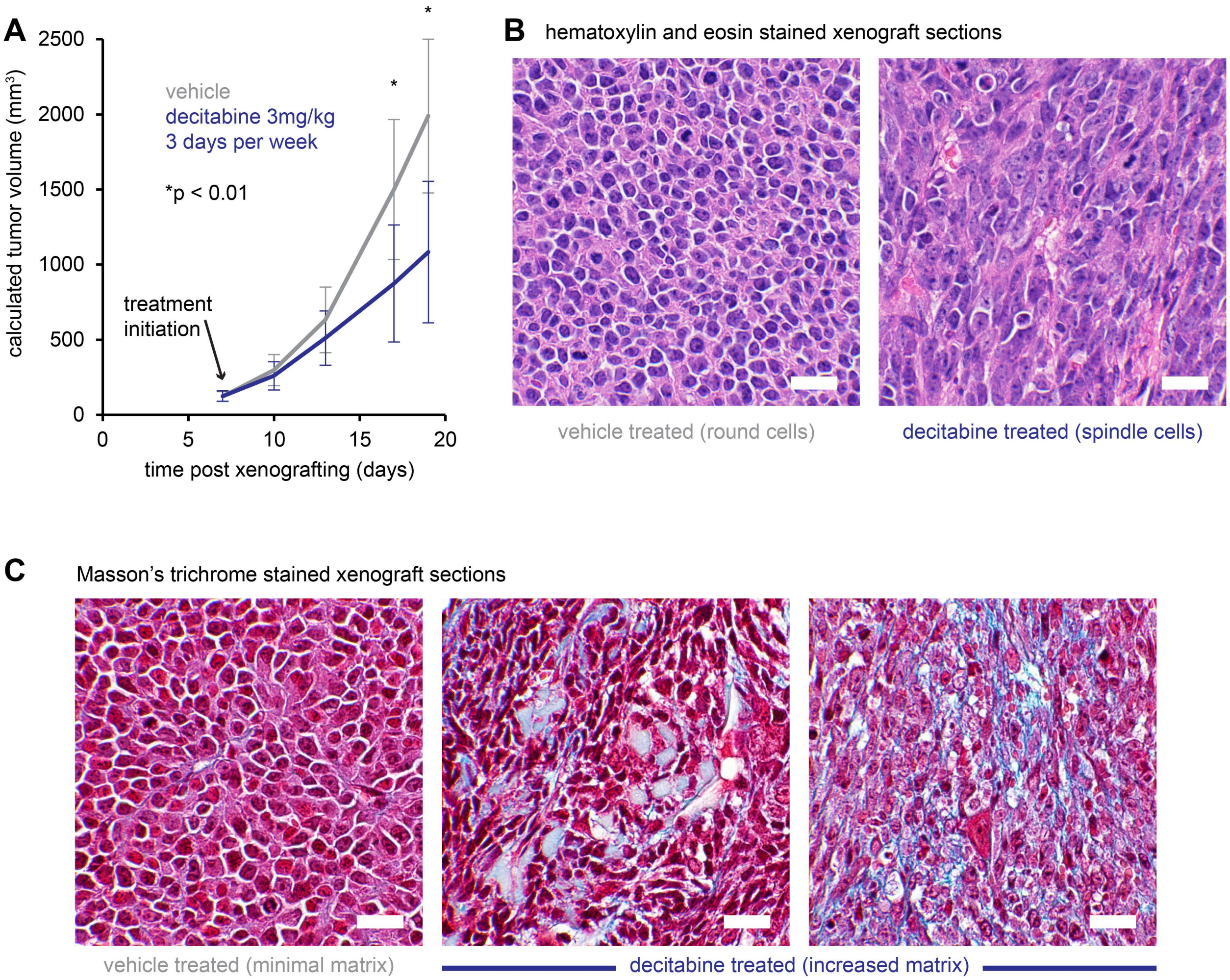


**Supplemental Figure 3. Decitabine slows growth and increases mesenchymal differentiation in SYO-1 cell line xenografts.** (**A**) Tumor growth curves for each group. Calculated tumor volumes are presented as mean ± SD. (n = 10 for both groups, but n = 8 for vehicle group on last two measurements, as tumors reached maximum size; *p*-values from two-tailed homoscedastic t-tests, with Bonferroni correction to $p < 0.01$.) (**B**) Photomicrographs of hematoxylin and eosin stained sections from the xenograft tumors after treatment, showing spindled histomorphology after decitabine. (**C**) Photomicrographs of Masson's trichrome stained sections of the SYO-1 xenografts, showing increased blue-staining collagen of the extracellular matrix after decitabine treatment. (All magnification bars are 20μm in length.)

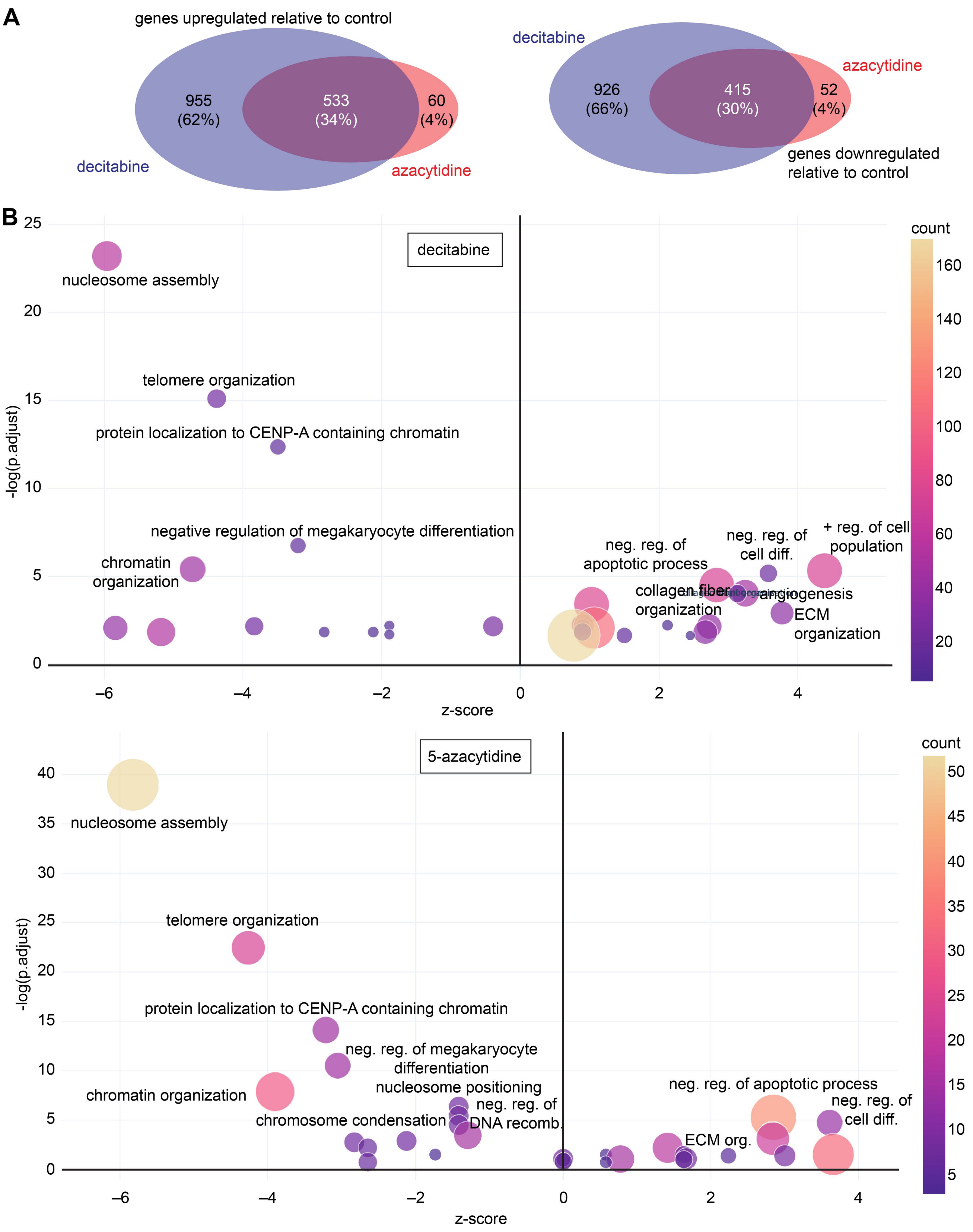


**Supplemental Figure 4. Demethylating agents impact transcription.** (**A**) Genes with log2 fold-change expression (<-1/>1) and adjusted $p < 0.05$ compared to control by decitabine and azacytidine in SYO-1 cells. (**B**) Bubble plots from GO analysis of up- and down-regulated genes in SYO-1 cells following decitabine or azacytidine treatment compared to control.

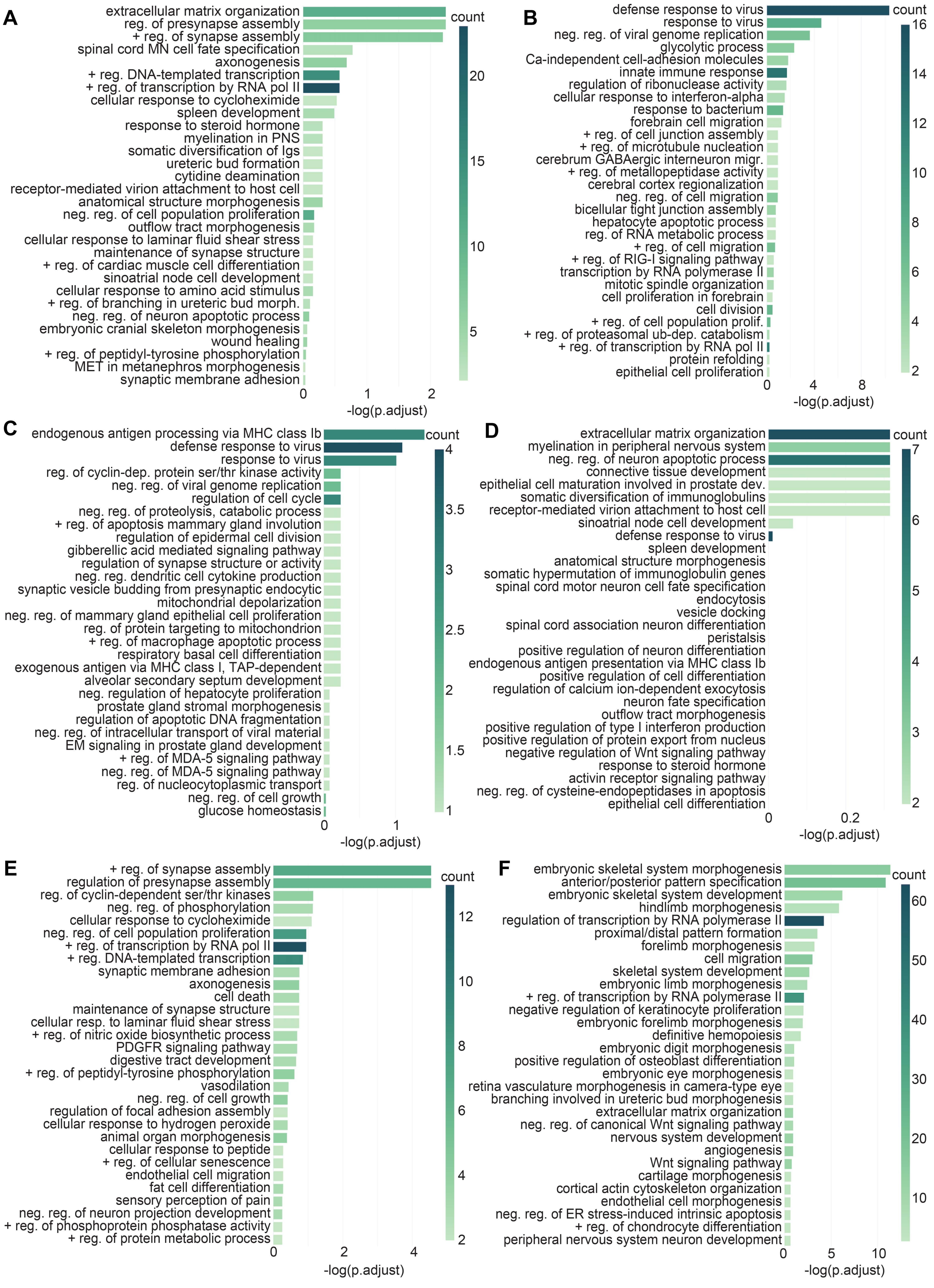
A
extracellular matrix organization
reg. of presynapse assembly
+ reg. of synapse assembly
spinal cord MN cell fate specification
axonogenesis
+ reg. DNA-templated transcription
+ reg. of transcription by RNA pol II
cellular response to cycloheximide
spleen development
response to steroid hormone
myelination in PNS
somatic diversification of Igs
ureteric bud formation
cytidine deamination
receptor-mediated virion attachment to host cell
anatomical structure morphogenesis
neg. reg. of cell population proliferation
outflow tract morphogenesis
cellular response to laminar fluid shear stress
maintenance of synapse structure
+ reg. of cardiac muscle cell differentiation
sinoatrial node cell development
cellular response to amino acid stimulus
+ reg. of branching in ureteric bud morph.
neg. reg. of neuron apoptotic process
embryonic cranial skeleton morphogenesis
wound healing
+ reg. of peptidyl-tyrosine phosphorylation
MET in metanephros morphogenesis
synaptic membrane adhesion
0
1
2
-log(p.adjust)
count
20
15
10
5
B
defense response to virus
response to virus
neg. reg. of viral genome replication
glycolytic process
Ca-independent cell-adhesion molecules
innate immune response
regulation of ribonuclease activity
cellular response to interferon-alpha
response to bacterium
forebrain cell migration
+ reg. of cell junction assembly
+ reg. of microtubule nucleation
cerebrum GABAergic interneuron migr.
+ reg. of metallopeptidase activity
cerebral cortex regionalization
neg. reg. of cell migration
bicellular tight junction assembly
hepatocyte apoptotic process
reg. of RNA metabolic process
+ reg. of cell migration
+ reg. of RIG-I signaling pathway
transcription by RNA polymerase II
mitotic spindle organization
cell proliferation in forebrain
cell division
+ reg. of cell population prolif.
+ reg. of proteasomal ub-dep. catabolism
+ reg. of transcription by RNA pol II
protein refolding
epithelial cell proliferation
0
4
8
-log(p.adjust)
count
16
14
12
10
8
6
4
2
C
endogenous antigen processing via MHC class Ib
defense response to virus
response to virus
reg. of cyclin-dep. protein ser/thr kinase activity
neg. reg. of viral genome replication
regulation of cell cycle
neg. reg. of proteolysis, catabolic process
+ reg. of apoptosis mammary gland involution
regulation of epidermal cell division
gibberellic acid mediated signaling pathway
regulation of synapse structure or activity
neg. reg. dendritic cell cytokine production
synaptic vesicle budding from presynaptic endocytic
mitochondrial depolarization
neg. reg. of mammary gland epithelial cell proliferation
reg. of protein targeting to mitochondrion
+ reg. of macrophage apoptotic process
respiratory basal cell differentiation
exogenous antigen via MHC class I, TAP-dependent
alveolar secondary septum development
neg. regulation of hepatocyte proliferation
prostate gland stromal morphogenesis
regulation of apoptotic DNA fragmentation
neg. reg. of intracellular transport of viral material
EM signaling in prostate gland development
+ reg. of MDA-5 signaling pathway
neg. reg. of MDA-5 signaling pathway
reg. of nucleocytoplasmic transport
neg. reg. of cell growth
glucose homeostasis
0
1
-log(p.adjust)
count
4
3.5
3
2.5
2
1.5
1
D
extracellular matrix organization
myelination in peripheral nervous system
neg. reg. of neuron apoptotic process
connective tissue development
epithelial cell maturation involved in prostate dev.
somatic diversification of immunoglobulins
receptor-mediated virion attachment to host cell
sinoatrial node cell development
defense response to virus
spleen development
anatomical structure morphogenesis
somatic hypermutation of immunoglobulin genes
spinal cord motor neuron cell fate specification
endocytosis
vesicle docking
spinal cord association neuron differentiation
peristalsis
positive regulation of neuron differentiation
endogenous antigen presentation via MHC class Ib
positive regulation of cell differentiation
regulation of calcium ion-dependent exocytosis
neuron fate specification
outflow tract morphogenesis
positive regulation of type I interferon production
positive regulation of protein export from nucleus
negative regulation of Wnt signaling pathway
response to steroid hormone
activin receptor signaling pathway
neg. reg. of cysteine-endopeptidases in apoptosis
epithelial cell differentiation
0
0.2
-log(p.adjust)
count
7
6
5
4
3
2
E
+ reg. of synapse assembly
regulation of presynapse assembly
reg. of cyclin-dependent ser/thr kinases
neg. reg. of phosphorylation
cellular response to cycloheximide
neg. reg. of cell population proliferation
+ reg. of transcription by RNA pol II
+ reg. DNA-templated transcription
synaptic membrane adhesion
axonogenesis
cell death
maintenance of synapse structure
cellular resp. to laminar fluid shear stress
+ reg. of nitric oxide biosynthetic process
PDGFR signaling pathway
digestive tract development
+ reg. of peptidyl-tyrosine phosphorylation
vasodilation
neg. reg. of cell growth
regulation of focal adhesion assembly
cellular response to hydrogen peroxide
animal organ morphogenesis
cellular response to peptide
+ reg. of cellular senescence
endothelial cell migration
fat cell differentiation
sensory perception of pain
neg. reg. of neuron projection development
+ reg. of phosphoprotein phosphatase activity
+ reg. of protein metabolic process
0
2
4
-log(p.adjust)
count
12
10
8
6
4
2
F
embryonic skeletal system morphogenesis
anterior/posterior pattern specification
embryonic skeletal system development
hindlimb morphogenesis
regulation of transcription by RNA polymerase II
proximal/distal pattern formation
forelimb morphogenesis
cell migration
skeletal system development
embryonic limb morphogenesis
+ reg. of transcription by RNA polymerase II
negative regulation of keratinocyte proliferation
embryonic forelimb morphogenesis
definitive hemopoiesis
embryonic digit morphogenesis
positive regulation of osteoblast differentiation
embryonic eye morphogenesis
retina vasculature morphogenesis in camera-type eye
branching involved in ureteric bud morphogenesis
extracellular matrix organization
neg. reg. of canonical Wnt signaling pathway
nervous system development
angiogenesis
Wnt signaling pathway
cartilage morphogenesis
cortical actin cytoskeleton organization
endothelial cell morphogenesis
neg. reg. of ER stress-induced intrinsic apoptosis
+ reg. of chondrocyte differentiation
peripheral nervous system neuron development
0
5
10
-log(p.adjust)
count
60
50
40
30
20
10

**Supplemental Figure 5. GO analysis of the biological processes in genes upregulated in mouse tumors.** (**A**) Analysis of 461 AZA group-only upregulated genes. (**B**) Analysis of 192 DAC-A-only upregulated genes. (**C**) Analysis of the 29 upregulated genes shared by the AZA and DAC-A groups. (**D**) Analysis of 320 AZA group-only genes. (**E**) Analysis of the 170 genes upregulated in common in both the AZA and DAC-B groups. (**F**) Analysis of 841 DAC-B group-only genes.

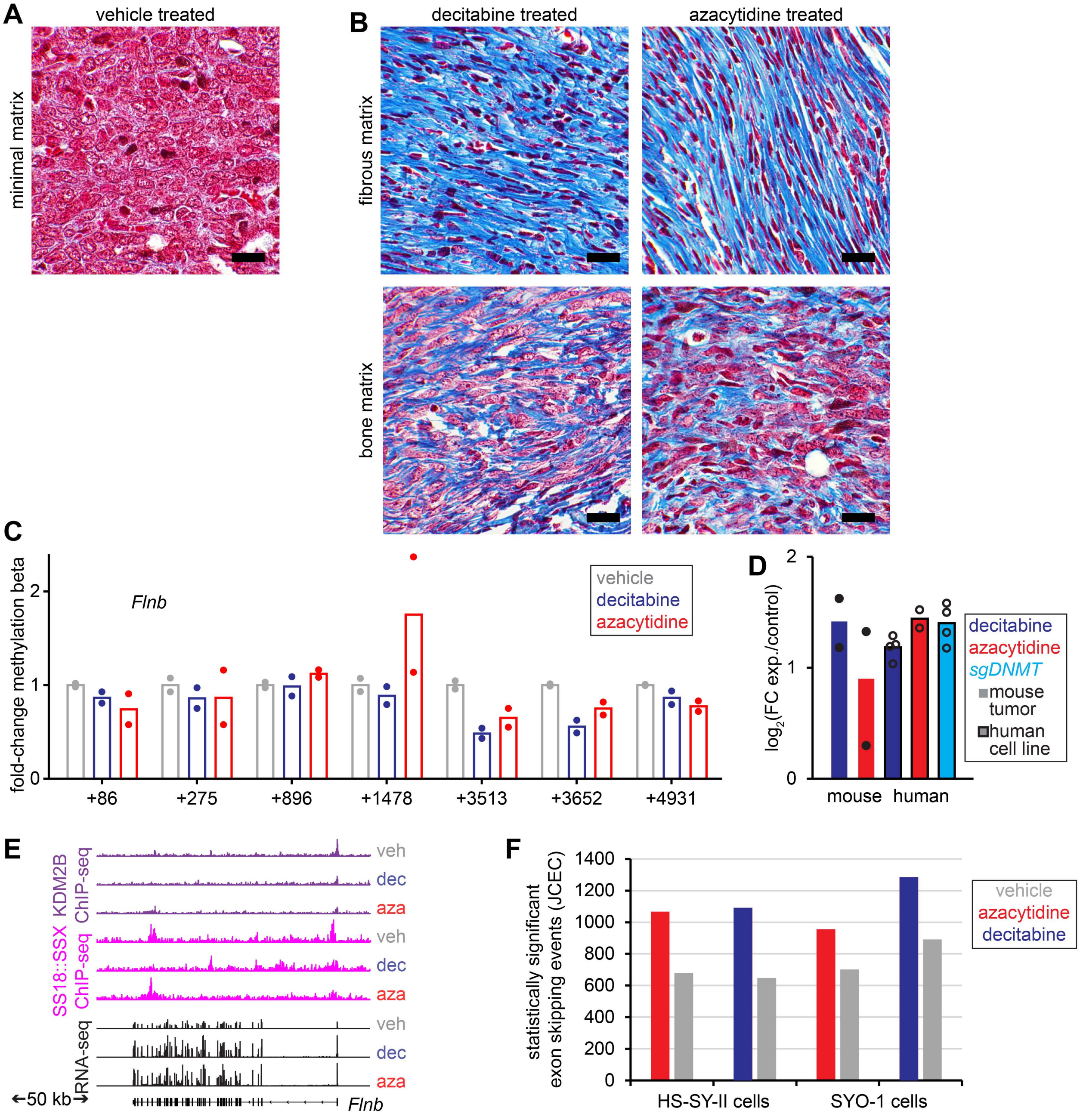


**Supplemental Figure 6. Pharmacological hypomethylation increases extra-cellular matrix production in SS.** (**A**) Photomicrographs of Masson's trichrome stained mouse SS tumor sections treated with vehicle or (**B**) azacytidine or decitabine, the latter showing blue stained collagen extracellular matrix and even some red stained matrix from partially calcified osteoid matrix. (**C**) Promoter methylation fold-changes for the *Flnb/FLNB* gene following vehicle, decitabine, or azacytidine treatments. (**D**) RNA-seq-determined log2-transformed fold-changes of expression of *Flnb/FLNB* over controls for decitabine, azacytidine, or *sgDNMT1*. (**E**) ChIP-seq tracks of KDM2B (0-1.76) and SS18::SSX (0-0.88), with RNA-seq (reads per million 0-4.36) for mouse SSs treated with vehicle, decitabine or azacytidine. (**F**) RNA-seq-determined exon skipping alternative splicing events in two human cell lines following azacytidine or decitabine, each compared to vehicle, calculated by the junction counts and exon counts (JCEC) method.
