# Supplementary figures and images for: DNA demethylating agents suppress preclinical models of synovial sarcoma
Source: J Clin Invest. 2025 Apr 29;135(13):e190855. doi: 10.1172/JCI190855 (PMC12208545; doi:10.1172/JCI190855)

Full unedited gel for Figure 2A

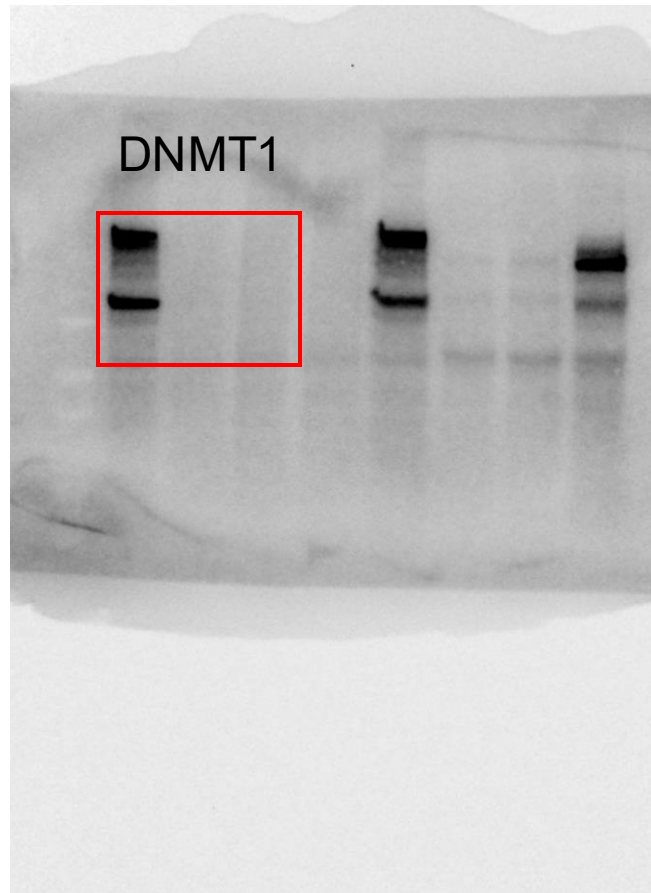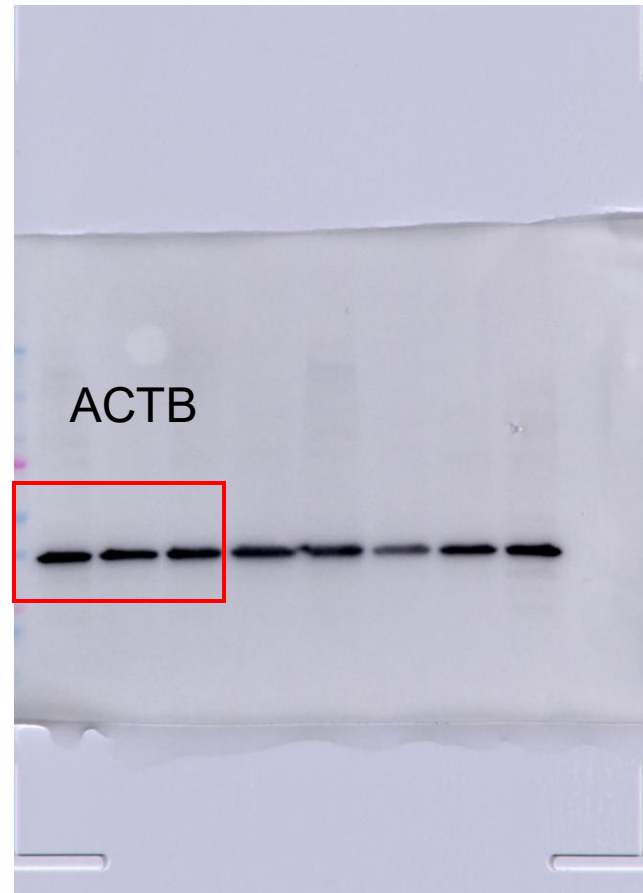

Supplement: Unedited blot and gel images [file jci-135-190855-s199.pdf]
